# Jasmonates induce Arabidopsis bioactivities selectively inhibiting the growth of breast cancer cells through CDC6 and mTOR

**DOI:** 10.1111/nph.17031

**Published:** 2020-11-19

**Authors:** Moritz Bömer, Imma Pérez‐Salamó, Hannah V. Florance, Deborah Salmon, Jan‐Hendrik Dudenhoffer, Paul Finch, Aycan Cinar, Nicholas Smirnoff, Amanda Harvey, Alessandra Devoto

**Affiliations:** ^1^ Department of Biological Sciences Plant Molecular Science and Centre of Systems and Synthetic Biology Royal Holloway University of London Egham TW20 0EX UK; ^2^ Natural Resources Institute University of Greenwich Central Avenue Chatham Maritime ME4 4TB UK; ^3^ Biosciences College of Life and Environmental Sciences University of Exeter Geoffrey Pope Building, Stocker Road Exeter EX4 4QD UK; ^4^ Institute of Environment, Health and Societies Brunel University London Kingston Lane Uxbridge UB8 3PH UK

**Keywords:** *Arabidopsis thaliana*, bioassay, cancer therapy, cell cycle, jasmonate, natural compounds

## Abstract

- Phytochemicals are used often *in vitro* and *in vivo* in cancer research. The plant hormones jasmonates (JAs) control the synthesis of specialized metabolites through complex regulatory networks. JAs possess selective cytotoxicity in mixed populations of cancer and normal cells.
- Here, direct incubation of leaf explants from the non‐medicinal plant *Arabidopsis thaliana* with human breast cancer cells, selectively suppresses cancer cell growth. High‐throughput LC‐MS identified Arabidopsis metabolites. Protein and transcript levels of cell cycle regulators were examined in breast cancer cells.
- A synergistic effect by methyljasmonate (MeJA) and by compounds upregulated in the metabolome of MeJA‐treated Arabidopsis leaves, on the breast cancer cell cycle, is associated with Cell Division Cycle 6 (CDC6), Cyclin‐dependent kinase 2 (CDK2), Cyclins D1 and D3, indicating that key cell cycle components mediate cell viability reduction. Bioactives such as indoles, quinolines and cis‐(+)‐12‐oxophytodienoic acid, in synergy, could act as anticancer compounds.
- Our work suggests a universal role for MeJA‐treatment of Arabidopsis in altering the DNA replication regulator CDC6, supporting conservation, across kingdoms, of cell cycle regulation, through the crosstalk between the mechanistic target of rapamycin, mTOR and JAs. This study has important implications for the identification of metabolites with anti‐cancer bioactivities in plants with no known medicinal pedigree and it will have applications in developing disease treatments

## Introduction

Plants produce many small molecules used as pharmaceuticals, insecticides, flavours and fragrances with commercial applications which derive from their common use in defence against biotic challenges (Pérez‐Salamó *et al*., 2019).

The ubiquitous plant stress hormone jasmonic acid (JA) and its oxylipin derivatives, such as methyljasmonate (MeJA) and jasmonate isoleucin (JA‐Ile), namely jasmonates (JAs) here, are potent regulators of plant defence, response to abiotic stress and developmental processes (Kazan, 2015; Riemann *et al*., 2015; Züst & Agrawal, 2017; Guo *et al*., 2018; Howe *et al*., 2018; Pérez‐Salamó *et al*., 2019). Environmental pressures induce endogenous JAs biosynthesis. JA signalling triggers complex responses in plant cells, including massive transcriptional and metabolic reprogramming, and defence proteins and protective specialized metabolites biosynthesis (Balbi & Devoto, 2008; Pauwels *et al*., 2009; Noir *et al*., 2013; Wasternack & Hause, 2013; Bömer *et al*., 2018; Pérez‐Salamó *et al*., 2019).

Jasmonates control specialized metabolites synthesis through complex gene regulatory networks to limit it to when necessary. JAs induce most classes of specialized metabolites, including alkaloids, terpenoids, glucosinolates and some phenylpropanoids (Balbi & Devoto, 2008; Zhou & Memelink, 2016; Pérez‐Salamó *et al*., 2019). The precursor of JA, cis‐(+)‐12‐oxophytodienoic acid (OPDA), also induces JA‐independent specialized metabolites (Wasternack & Hause, 2013). Primary and secondary sulfur‐related pathways leading to the synthesis of glucosinolates, have been shown to be MeJA‐responsive in Arabidopsis (Jost *et al*., 2005). Moreover the production of several agricultural and medicinal compounds, including glucosinolates, occurs through tryptophan metabolism (Smolen *et al*., 2002). The cabbage (Brassica) family, which includes *Arabidopsis thaliana*, is a rich source of glucosinolates and most biological activities for these in both plants and animals, reside with their cognate hydrolytic products. The isothiocyanates, such as sulforaphane, are outstanding examples (Dinkova‐Kostova & Kostov, 2012).

Humans have long used plant‐derived specialized metabolites as phytopharmaceuticals. Many phytochemicals have been identified as bioactive, including the prominent JA‐induced anticancer drug, taxol (Baldi & Dixit, 2008). Fingrut & Flescher (2002) showed that JAs are potential anticancer agents (Fingrut & Flescher, 2002). JAs showed selective cytotoxicity in mixed populations of cancer and normal cells from chronic lymphocytic leukaemia patients (Fingrut & Flescher, 2002; Flescher, 2007). MeJA‐induced apoptotic death in cancer cells and the survival rates of mice bearing lymphoma were higher following MeJA treatment (Fingrut & Flescher, 2002). JAs and synthetic analogues exhibit anticancer activity in human breast, cervix, colon, colorectal, gastric, hepatoma, lung, lymphoma, melanoma, myeloid leukaemia, neuroblastoma, prostate and sarcoma cancer cells (Balbi & Devoto, 2008; Cesari *et al*., 2014; Pérez‐Salamó *et al*., 2019).

Three different mechanisms of action – bioenergetic, re‐differentiation and reactive oxygen species (ROS)‐mediated mechanisms – were proposed to explain the activity of JAs against cancer cells (Flescher, 2007). MeJA has powerful anticancer activities both *in vitro* and *in vivo* (Fingrut & Flescher, 2002; Rotem *et al*., 2003; Fingrut *et al*., 2005; Flescher, 2007; Cohen & Flescher, 2009; Elia & Flescher, 2013; Li *et al*., 2017; Peng & Zhang, 2017). JAs induce both apoptotic and nonapoptotic cancer cell death, independent of their p53 status, acting directly and selectively on mitochondria in cancer cells (Fingrut *et al*., 2005; Rotem *et al*., 2005). MeJA causes bioenergetic dysregulation and cell cycle arrest in different cancer cell types (Rotem *et al*., 2005; Li *et al*., 2017). MeJA treatment causes G0/G1 and S‐phase arrest and induces apoptosis by increasing expression of tumour necrosis factor receptor 1 (TNFR1), activation of mitogen‐activated protein kinase (MAPK) and caspase‐8, and decreasing the mitochondrial membrane potential in MCF‐7 breast cancer cells (Yeruva *et al*., 2008). In non‐small cell lung cancer cells, MeJA induces apoptosis (Zhang *et al*., 2016) and exerts its anticancer activity through downregulation of enhancer of zeste 2 polycomb repressive complex 2 subunit (EZH2), a histone methyltransferase, and the catalytic subunit of polycomb repressive complex 2 (PRC2) (Fu *et al*., 2014). Taken together, these findings suggest that some of MeJA's anticancer activities are mediated by compounds upregulated by MeJA, although so far, the mechanisms of action of JAs and their induced metabolites on cancer cells, have never been compared.

Here, direct incubation of leaf explants of the nonmedicinal plant *Arabidopsis thaliana* with human breast cancer cells is established in a bioassay comparing the efficacy of JA‐regulated, specialized metabolites and MeJA on breast cancer cell lines. Metabolite extracts derived directly from the bioassay, including media and cancer cell controls, as well as wild‐type and mutant plants, proved to be effective in the search for plant‐derived, JA‐induced specialized metabolites with anticancer activities. This system demonstrated consistently, the biological activity of plant material subjected to JA treatment on the growth inhibition of breast cancer cells. Arabidopsis mutants allowed dissection of the plant mechanisms controlling these bioactivities. The bioactivity of MeJA‐treated, Arabidopsis leaf samples on the growth of breast cancer cells was Coronatine‐insensitive protein 1 (COI1)‐dependent and mediated by JA‐induced plant‐derived specialized metabolites such as indoles, quinolines and OPDA. The inhibitory effect was far superior to that of MeJA alone. Clustering and *in silico* identification of plant‐derived MeJA‐induced and COI1‐dependent metabolic features showed that the effects on breast cancers cells are unlikely to be ascribed to individual features and that cancer cells metabolism affects bioactivity. We showed that the post‐translational downregulation of CDC6, CDK2, Cyclin D1 and Cyclin D3 is part of the mechanism to reduce breast cancer cell viability. Our analysis supports conservation, across kingdoms, of the regulation of the cell cycle through crosstalk between the mechanistic target of rapamycin, mTOR and JAs.

## Materials and Methods

### Plant leaf disk bioassay using human breast cancer cells

The antiproliferative effect of Arabidopsis plants aged 11 d after sowing (DAS) ±50 µM methyljasmonate (MeJA) for 24 h was evaluated using the 3‐(4,5‐dimethylthiazol‐2‐yl)‐2,5‐diphenyltetrazolium bromide (MTT) cell viability assay.

MDA‐MB‐361, T‐47D and MCF‐10A were seeded in 96‐well plates with 15 000 cells per well in a final volume of 100 µl medium, and were left to set overnight. After 24 h, 3 × 1 mm (diameter) leaf disks excised from the first pair of true leaves were added aseptically to each well using a 1‐mm Sample corer (InterFocus, Cambridge, UK) and co‐incubated with the cells for 72 h. Relative quantification of the cell proliferation of the human breast cancer cell lines T‐47D and MDA‐MB‐361 and the nontumourigenic cell line MCF‐10A was assessed by MTT assay. For all treatments, the leaf disks and culture media were removed after the 72 h incubation period and the MTT reagent was added to the wells in fresh medium. The cytotoxic effects of MeJA on the cells were evaluated using a trypan blue inclusion assay.

### Western blotting

Total protein was extracted from the cell pellets using the NucleoSpin RNA/Protein Kit (Macherey‐Nagel, Dueren, Germany), and concentration determined using the Protein Quantification Assay Kit (Macherey‐Nagel) and a microplate photometer (Multiskan EX, Thermo Scientific, Waltham, MA, USA). Equal amounts of protein (10 μg) were resolved by SDS‐PAGE. Band intensity, mirroring protein levels were visualized using chemiluminescence detection systems Supersignal West Pico (Thermo Scientific) or Substrat HRP Immobilon Western (Merck Millipore) following the manufacturers’ instructions.

### Cell cycle analysis

Ploidy levels were determined by flow cytometry using a Ploidy Analyser PAS (Sysmex Partec GmbH, Münster, Germany), with UV excitation at 366 nm from a mercury arc lamp. Nuclei were released using Cystain extraction buffer, filtered through a Cell trics filter and stained with Cystain fluorescent buffer (Partec). At least fifteen thousand nuclei were used for each ploidy measurement and the percentages of cells in the different phases of the cell cycle was calculated.

### Quantitative real‐time (qRT)‐PCR

Analysis of total RNA yield was performed on a nanodrop spectrophotometer (Labtech, Heathfield, UK). cDNA preparation was performed using the QuantiTect Reverse Transcription kit (Qiagen). Real‐time amplification was performed using SYBR Green JumpStart (Sigma‐Aldrich) according to the manufacturer's instructions. Transcript analysis was performed from RNA samples derived from at least five independent experiments. The primer sequences are listed in Supporting Information Table S1.

### Metabolite profiling by LC‐MS/MS

Metabolite profiling was performed using a QToF (Quadrupole Time of Flight) 6520 mass spectrometer (Agilent Technologies, Palo Alto, CA, USA) coupled to a 1200 series Rapid Resolution HPLC system.

### Data extraction and processing

The raw data files (Agilent *.d) of leaf disc‐containing samples were processed with mass profiler (v.B.08.00; Agilent) to extract features of interest (FOIs) using the built‐in molecular feature extraction algorithm. Differentially expressed features were identified by three‐way ANOVA (*P* < 0.05) using the Benjamini–Hochberg multiple comparison correction. This list was used for cluster and heat map generation. To lead the discovery of jasmonic acid (JA)‐regulated specialized metabolites with potential in inhibiting human breast cancer cell growth, we ran linear regression models on the normalized (zero mean and unit variance) log_2_‐transformed abundances of each metabolite (total of 1757) with subsequent tests of *a priori* defined treatment contrasts (Hothorn *et al*., 2008). These tests served as filtering conditions and metabolites that met them, were aggregated into corresponding sets (Table 1). All tests were performed with a significance level of *P* = 0.05.

**Table 1:** *A priori* defined contrasts used as filtering conditions for the discovery of metabolites inhibiting breast cell cancer growth.

Treatment contrast	Cell background	Set	Number of metabolites	Overlap	Features of interest
Col *gl1* +MeJA > Col *gl1* −MeJA	T‐47D	A1	236	73	41^a^
Col *gl1* +MeJA > Col *gl1* −MeJA	No cell control	A2	134		
Col *gl1* +MeJA > *coi1*‐16B + MeJA	T‐47D	B1	427	262	
Col *gl1* +MeJA > *coi1*‐16B +MeJA	No cell control	B2	341		
Col *gl1* −MeJA = *coi1*‐16B −MeJA	T‐47D	C1*	1188	1040	
Col *gl1* −MeJA = *coi1*‐16B −MeJA	No cell control	C2*	1251		
*coi1*‐16B +MeJA > *coi1*‐16B −MeJA	T‐47D	D1	110	30	10^b^
*coi1*‐16B +MeJA > *coi1*‐16B −MeJA	No cell control	D2	78		
*coi1*‐16B +MeJA > Col *gl1* +MeJA	T‐47D	E1	345	207	
*coi1*‐16B +MeJA > Col *gl1* +MeJA	No cell control	E2	268		
Col *gl1* −MeJA +T‐47D > Col *gl1* −MeJA −T‐47D		F1	264	187	117^c^
Col *gl1* +MeJA +T‐47D > Col *gl1* +MeJA −T‐47D		F2	292		
*coi1*‐16B −MeJA +T‐47D > *coi1*‐16B −MeJA −T‐47D		F3	251	192	
*coi1*‐16B +MeJA +T‐47D > *coi1*‐16B +MeJA −T‐47D		F4	277		
Col *gl1* −MeJA +T‐47D < Col *gl1* −MeJA −T‐47D		G1	298	141	98^d^
Col *gl1* +MeJA +T‐47D < Col *gl1* +MeJA −T‐47D		G2	250		
*coi1*‐16B −MeJA +T‐47D < *coi1*‐16B −MeJA −T‐47D		G3	350	247	
*coi1*‐16B +MeJA +T‐47D < *coi1*‐16B +MeJA −T‐47D		G4	292		

COI1, Coronatine‐insensitive protein 1; Col GL1, Trichome differentiation protein (WT); MeJA, methyljasmonate.

^a^ *COI1*‐dependent MeJA‐induced in Col *gl1*.

^b^ MeJA‐induced in *coi1*‐16B.

^c^ More abundant in T‐47D background (end‐ or by‐products of T‐47D metabolism).

^d^ Less abundant or absent in T‐47D background (metabolized cancer cell media).

* Metabolites in sets C1 and C2 met the condition if metabolite abundances were not significantly different at *P* = 0.05.

For further analysis, including medium only and medium plus T‐47D cells, the raw data files were aligned and subjected to recursive molecular feature extraction using profinder (v.B.10.00; Agilent Technologies, Santa Clara, CA, USA). The resulting set of compounds were exported to massprofiler professional (Agilent Technologies) and analyzed to identify plant‐specific and MeJA‐induced features.

Where available, MS/MS spectra of FOIs were extracted from raw data files using masshunter qualitative analysis software (v.B07.00; Agilent Technologies) and compared with MS/MS data from Metlin and MassBank to provide putative identifications. The identification of JA and cis‐(+)‐12‐oxophytodienoic acid (OPDA) was further confirmed by comparison of retention time and spectra with standards. The stereoisomers of these compounds were not resolved by the chromatographic method used. Predicted MS/MS spectra were generated with the MetFrag tool (https://msbi.ipb‐halle.de/MetFrag) (Ruttkies *et al*., 2016).

The data integral to the paper (fully documented LC‐MS/MS analysis) is available through https://royalholloway.figshare.com/, doi: 10.17637/rh.13079153.

For further details and information on plant materials, human breast cancer cell lines, treatment, antibodies and analysis, see Supporting Information Methods S1.

## Results

### Methyljasmonate inhibits the growth of breast cancer but not that of nontumorigenic cells

**Fig. 1:**
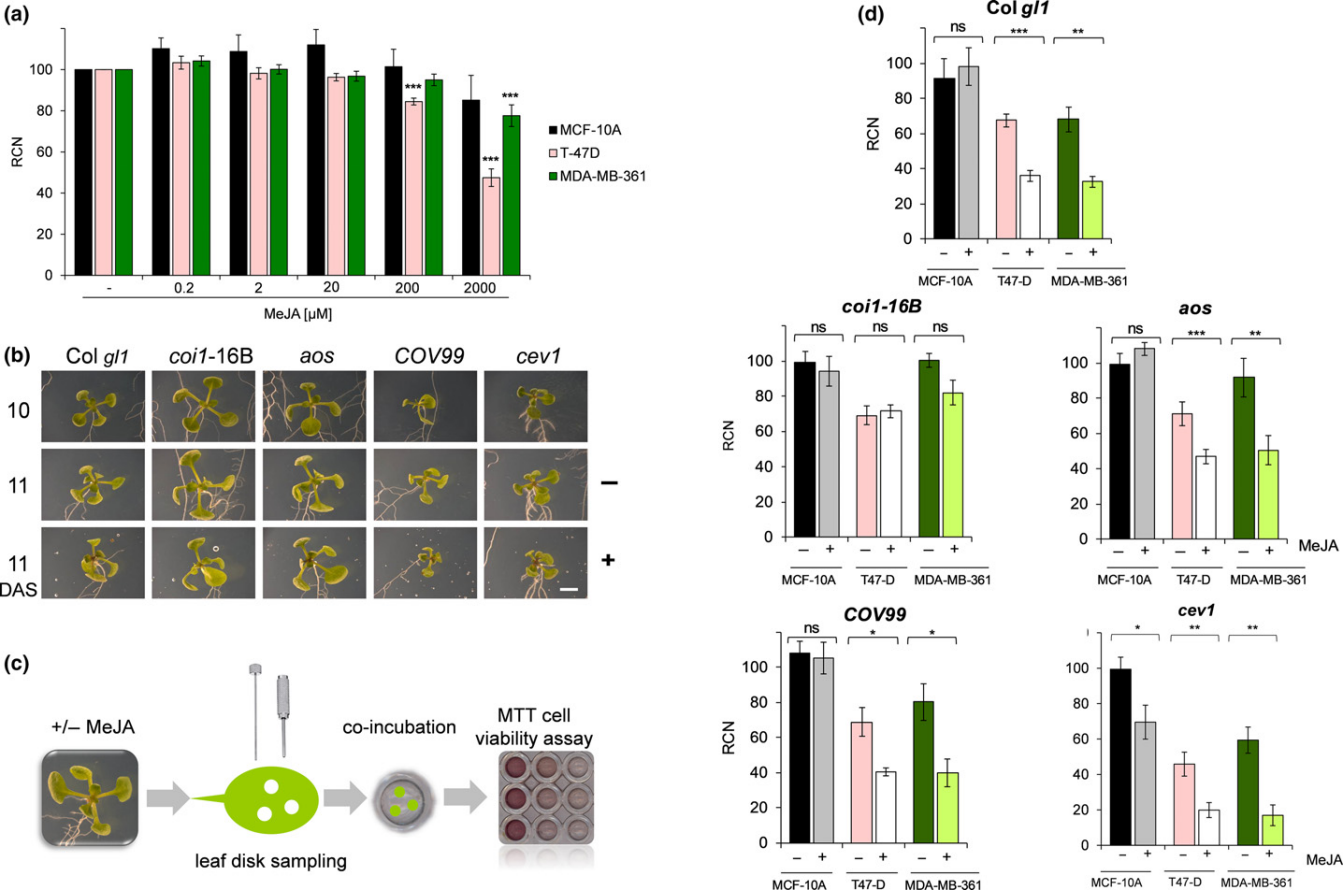
The effects of methyljasmonate (MeJA) treatment or co‐culture of Arabidopsis jasmonic acid (JA) mutants on human breast cancer cells T‐47D and MDA‐MB‐361, and on nontumourigenic MCF‐10A. (a) Effect of MeJA treatment on the growth of cancer T‐47D, MDA‐MB‐361 or nontumourigenic MCF‐10A cell lines. The data quantified by 3‐(4,5‐dimethylthiazol‐2‐yl)‐2,5‐diphenyltetrazolium bromide (MTT) assay are presented as as relative cell number (RCN) compared to the vehicle (ethanol) control (−). (b) Plants were grown vertically *in vitro* and photographed at 10 d after sowing (DAS) before transferring to plates containing media ± 50 µM MeJA. Plants treated (+) and untreated (−) were photographed again after 24 h of treatment at 11 DAS to visualize the rosette phenotype. Bar, 5 mm. (c) At 11 DAS three 3‐mm leaf disks were excised and co‐incubated in three or four replicate wells with cancer or nontumourigenic cells for 72 h after which an MTT assay was performed. (d) Effect of the co‐incubation of MeJA‐treated (+) and untreated (−) plant leaf explants from wild‐type (WT) background Col gl1 or JA mutants *coi1*‐16B, *aos*, *COV99* and *cev1* on the growth of cancer T‐47D, MDA‐MB‐361 or nontumourigenic MCF‐10A cell lines. The data quantified by MTT assay are presented as RCN of viable cells as a % compared to the growth control. (a, c) Bars correspond to the mean of at least three independent experiments (error bars denote the SEM). The number of asterisks denote significance values against the untreated control (−) of each genotype using two‐tailed Student’s *t*‐test: *, *P* < 0.05; **, *P* < 0.01; ***, *P* < 0.001; ns, not significant. For detailed *P*‐values see Supporting Information Table S2.

The activity of MeJA was compared in human breast cancer cell lines T‐47D and MDA‐MB‐361, with the nontumourigenic mammary cell line MCF10A (Fig. 1a). Both T‐47D and MDA‐MB‐361 cell lines are ductal and oestrogen receptor (ER) positive with MDA‐MB‐361 expressing HER2 (Keydar *et al*., 1979). Previously, low or no JA‐induced cytotoxicity in healthy cells were reported (Fingrut & Flescher, 2002; Rotem *et al*., 2003, 2005; Reischer *et al*., 2007; Tong *et al*., 2008).

Relative quantification of cell numbers, in response to increasing MeJA concentrations (according to Cesari *et al.*, 2014) was assessed, and dose–response curves obtained. Concentrations of 200 µM and 2 mM MeJA significantly inhibited T‐47D cells growth. MDA‐MB‐361 cell growth also was significantly suppressed at 2 mM MeJA, but less than T‐47D (relative cell number (RCN) 78% and 47%, respectively). The leaf disks and culture media were removed after the 72 h incubation period and the MTT reagent was added to the wells in fresh medium to avoid interference of any compound released by the leaf explants or MeJA that could have led to the reduction of MTT to formazan (through differential regulation of enzyme activity). The effects seen are therefore a direct result of changes in cell number rather than in enzyme activity. The dose–response curves were used to calculate the half maximal inhibition concentration (IC_50_) values and these were determined as 1.87, 4.44 and 5.14 mM for T‐47D, MDA‐MB‐361 and MCF‐10A, respectively.

The effect of MeJA on cell cycle progression was determined by flow cytometry: treatment resulted in T‐47D cell cycle arrest in G0/G1 (Fig. S1B–E). Nontumourigenic MCF‐10A cells were not significantly affected by MeJA or treated Arabidopsis (Figs 1a, S1A–E). Cell death also was only mildly affected at concentrations ≥ 200 µM (Fig. S1A), demonstrating that MeJA had more profound effects on survival of the tumour cell lines.

### A bioassay of activity of leaf disks on different breast cancer and nontumorigenic cell types

We assayed the effects of MeJA treatment of Arabidopsis leaves before explants were taken, on the growth of breast cancer cells T47‐D and MDA‐MB361, and on nontumourigenic MCF10‐A. *Arabidopsis thaliana* mutants impaired in JA biosynthesis or signal perception were tested (Fig. 1b–d): the *coi1*‐16B mutant for the JA receptor Coronatine‐insensitive protein 1 (COI1), displaying a JA‐insensitive phenotype (Ellis & Turner, 2002; Westphal *et al*., 2008; Noir *et al*., 2013); the transgenic COI1 overexpressor (COV99), overexpressing line the JA receptor COI1 (Devoto *et al*., 2002; Bömer *et al*., 2018); the CONSTITUTIVE EXPRESSION OF VSP1 (*cev*), with higher concentrations of JAs (Ellis & Turner, 2001); and the allene oxide synthase (*aos*) knock‐out mutant, defective in the JA biosynthetic gene CYP74A (AOS) (Park *et al*., 2002), unable to produce JAs but capable of JA responses. All JA mutants except for *coi1*‐16B showed a clear phenotypic response to the 24 h MeJA treatment including some visible effects on the growth (Fig. 1b), previously associated with JA treatment (Shan *et al*., 2009; Noir *et al*., 2013). The effectiveness of the JA treatment also was confirmed through the expression of JA‐responsive genes such as *Vegetative storage protein* (*VSP*) 1 and *2* and *AOS* (data not shown). T‐47D cells, treated with MeJA or wild‐type (WT) Arabidopsis were observed under bright‐field microscopy (Fig. S2). Incubation with untreated leaf disks visibly decreased cell density, inducing rounder morphology and increasing floating debris. These changes were consistent with increased cell death (Fig. S1A).

The use of a bioassay originally devised to analyze chemopreventive glucosinolates in murine hepatoma cells (Wang *et al*., 2002), was extended to human cancer cell lines and further modified to test cell viability with MTT. Direct analysis of the effects of single leaf disks from the Arabidopsis Col *trichome differentiation protein (gl1)* (WT) or mutants was performed (Fig. 1c). Suppression of tumour cell growth was consistently the strongest when cells were co‐incubated with MeJA‐treated plant samples compared to the untreated control plant disks, except for *coi1*‐16B (Fig. 1d). Arabidopsis explants treated with 50 µM MeJA showed an inhibitory effect comparable to the treatment with the highest (mM) concentrations of MeJA (Fig. 1a,d). Both T‐47D and MDA‐MB‐361 cancer cell lines showed comparable responses (Fig. 1d). RCN inhibition values for MeJA‐treated plant leaf samples compared to the untreated controls are shown in Table S2. The growth of T‐47D was reduced to 68% RCN when exposed to untreated WT disks, but was significantly more reduced (36% RCN, *P* < 0.001) when exposed to MeJA‐treated disks (Fig. 1d). This compares to 68% and 33% RCN (respectively) for MDA‐MB‐361 cells. Mutant *aos* reduced the cell growth of T‐47D to 71% and 47% RCN for untreated and MeJA‐treated plant leaf disks, respectively. This compares to 92% and 50% RCN (respectively) for MDA‐MB‐361 cells (Table S2). The *aos* RCN inhibition values of 33% for T‐47D and 46% for MDA‐MB‐361 cells were slightly, but not significantly, lower when compared to WT (Table S2). A WT‐like effect was exerted by *COV99* samples with 69% and 41% RCN for untreated and treated disks (respectively) when testing T‐47D cells, and 80% and 40% (respectively) for MDA‐MB‐361. The inhibition of both breast cancer cell lines by *cev1* untreated and treated leaf disks was higher than that caused by WT. RCN values reached 46% and 20%, and 59% and 17% for untreated and treated samples when testing T‐47D and MDA‐MB‐361 cells, respectively. Consequently, inhibition values of *cev1* samples also were found to be higher compared to WT samples (Table S2), consistent with the presence of elevated concentrations of endogenous JAs. For all plant samples tested, co‐incubation with excised leaf disks resulted in significantly lower RCN values when testing the growth of T‐47D and MDA‐MB‐361 cancer cells. Remarkably, the growth of nontumourigenic MCF‐10A cells was not significantly affected, except for MeJA‐treated *cev1,* which has constitutive JA responses (Ellis & Turner, 2001).

The differential effect between MeJA‐treated and untreated plant samples was significant for all Arabidopsis mutant lines tested except for *coi1*‐16B (Fig. 1d). MeJA‐treated *coi1*‐16B samples did not reduce the cell growth of either cancer cell line further when compared to the untreated *coi1*‐16B controls, showing that the observed differential effect between MeJA‐treated and untreated leaf samples on the breast cancer cell growth was *COI1*‐dependent, as reflected in significantly lower inhibition values compared to the Col *gl1* WT (Table S2). We also tested the effect of Arabidopsis mutants impaired in glucosinolates or tryptophan metabolism (Bender & Fink, 1998; Barth & Jander, 2006; Bednarek *et al*., 2009) on T47‐D and MCF10A cells (Fig. S3; Table S2). These mutants showed no obvious or significant differential effects on the growth of the T‐47D or the nontumourigenic MCF‐10 lines compared to their Col‐0 WT.

In summary, the results obtained for *coi1*‐16B were consistent with the role of *COI1* in the JA signalling pathway. However, higher expression of the JA receptor in *COV99* (Bömer *et al*., 2018) did not cause any additional effects. The mutant *aos*, lacking the positive feedback loop amplification of the JA signal (Park *et al*., 2002), showed a less pronounced, although not significantly different, effect compared to the WT. In accordance with the JA‐dependency of the observed effects on breast cancer cells, *cev1*, which has constitutive JA responses (Ellis & Turner, 2001), displayed the strongest inhibitory potential.

### Metabolite profiling of human breast cancer T‐47D cell culture media after incubation with Arabidopsis

**Fig. 2:**
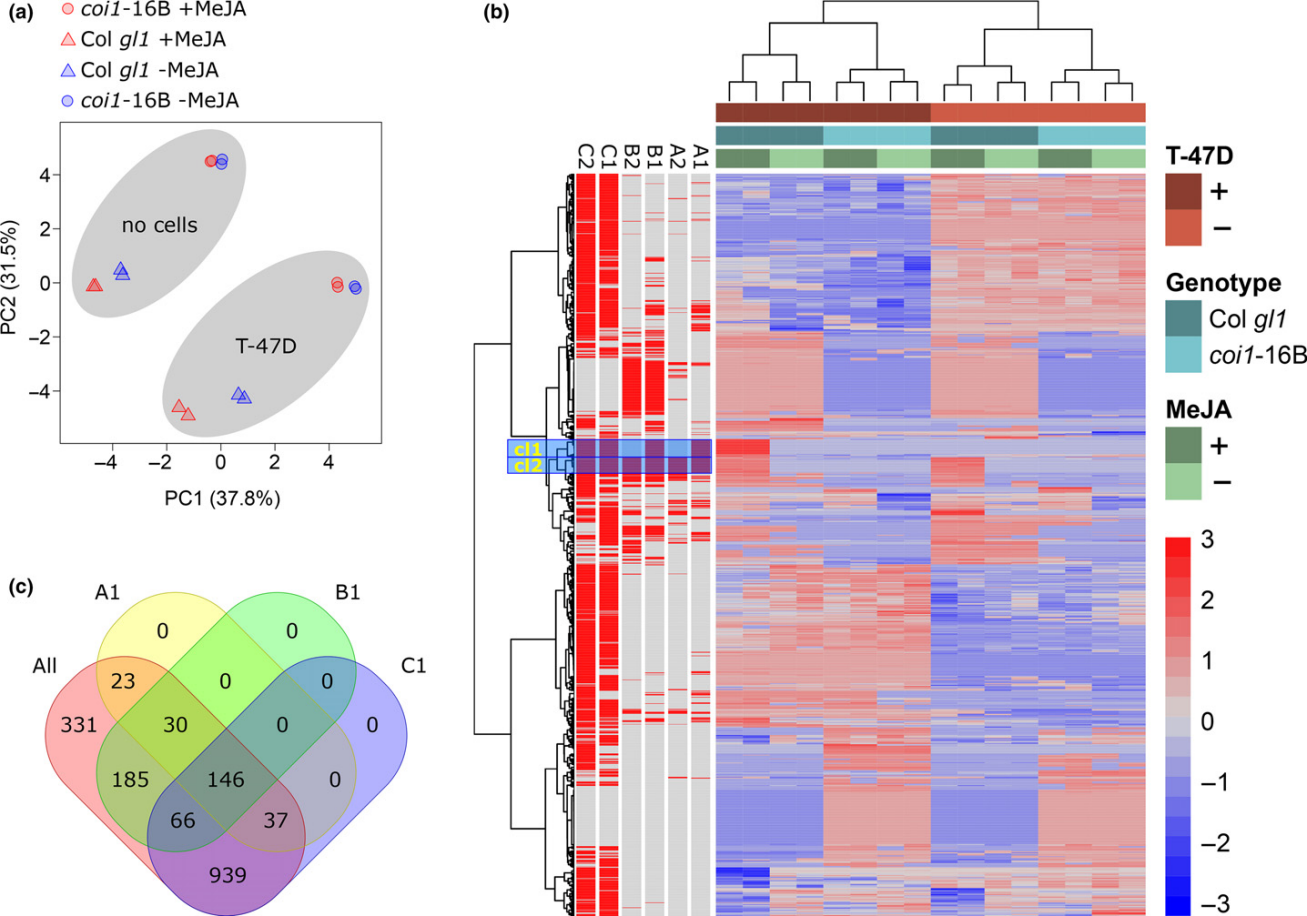
Comparative metabolite analysis of Arabidopsis plant leaf disk bioassay. (a) Principal component analysis showing discrimination of T‐47D and no cells background, Arabidopsis wild‐type (WT) and *coi1*‐16B plant leaf disks, and methyljasmonate (MeJA) treatment regime, based on metabolite profiles. (b) The heat map shows normalized abundances of all detected chemical features. Samples and chemical features (putative metabolites) were clustered based on the Euclidean distances of their normalized metabolite abundances using Ward's (clustering) algorithm. Sets A1, A2, B1, B2, C1 and C2 show the chemical features that match (red bars) the conditions of the filtering analysis (A, Col *gl1* + MeJA > Col *gl1*−MeJA; B, Col *gl1* + MeJA > *coi1*‐16B + MeJA; C, Col *gl1*−MeJA = *coi1*‐16B −MeJA; 1, T‐47D background; 2, no cell controls; *P* = 0.05; see Table 1 for details and number of metabolites meeting the conditions). (c) Venn diagram of common features matching filtering conditions in T‐47D background, where 146 features of interest were identified, most of which are part of the two highlighted clusters cl1 and cl2 in the heat map. R scripts used to analyze data and to generate the figures are provided as Supporting Information (Notes S1).

High‐throughput metabolic profiling to investigate the effects of JAs on Arabidopsis plants in isolation has been performed previously, albeit in different mutants and experimental conditions, on leaf intracellular extracts, and multivariate statistical analyses performed to obtain compound libraries (Cao *et al.*, 2016). Active compounds from plants need identification and mechanisms of action characterized to assess the full potential of the bioactives for clinical trials and applications, efficiency and any adverse effects. The inhibitory effects of MeJA‐treated Arabidopsis explants on human breast cancer cell growth, encouraged a search for specialized metabolites using untargeted LC‐MS/MS metabolic profiling. Candidate bioactive MeJA‐inducible compounds were predicted to be more abundant in media of MeJA‐treated WT treatments than with *coi1*‐16B. We focussed our analysis on compounds present in the cell media that could be recognized by surface receptors. Molecular feature extraction using massprofiler identified co‐eluting isotopes and adducts comprising 1757 putative features of interest (FOIs) in positive and negative ion modes (Table S3). Principal component analysis (PCA) based on the relative abundance of these FOIs showed clear discrimination between (1) the T‐47D and no cell backgrounds; (2) the Arabidopsis WT and *coi1*‐16B plant leaf disks; and (3) the MeJA treatment in the Col *gl1* samples (Fig. 2a). Hierarchical clustering of all FOIs based on their normalized metabolite abundances resulted in well‐defined clusters (Fig. 2b). Distinct metabolite profiles could be detected in the incubation medium from different treatments. The individual features were further filtered using predefined conditions (Table 1), identifying 146 FOIs (Fig. 2c) which responded to MeJA in Col *gl1* (A1) more than in *coi1*‐16B (B1) and were likewise abundant in untreated Col *gl1* compared to untreated *coi1*‐16B (C1). These features fell into clusters 1 and 2 (Fig. 2b) representing compounds affecting the viability of the T‐47D cells. Clusters 1 and 2 identified 69 FOIs present or absent in the no T‐47D cell control samples, respectively. Cluster 2 FOIs were *COI1*‐dependent, MeJA‐induced and produced in the Col *gl1* WT background independently of the presence or absence of T‐47D cancer cells and, therefore, plant‐derived. By contrast, Cluster 1 FOIs were *COI1*‐dependent, MeJA‐induced and occurred in the Col *gl1* WT background, but only in the presence of T‐47D cancer cells. Further filtering (Table 1) identified FOIs MeJA‐induced in *coi1*‐16B (10 FOIs), and those more/less abundant in the T‐47D cell background. These are likely to represent end‐ or by‐products of T‐47D metabolism (117 FOIs), compared to cancer cell media compounds, metabolized by the T‐47D cells (98 FOIs), respectively.

Further analysis identified plant derived Col *gl1*‐specific MeJA‐induced features. All samples, including medium‐only and medium plus T‐47D cells were re‐aligned and molecular feature extraction using masshunter profinder followed by statistical analysis with massprofiler professional, performed. This analysis extracted 161 and 105 putative features in positive and negative ion mode respectively which were present exclusively in samples containing leaf discs (Table S4). Of these plant‐specific features, 21 (15 and six in positive and negative ion modes, respectively) were induced by MeJA in Col *gl1* but not in *coi1*‐16B (Table S4).

### Discovery of bioactive metabolites

**Fig. 3:**
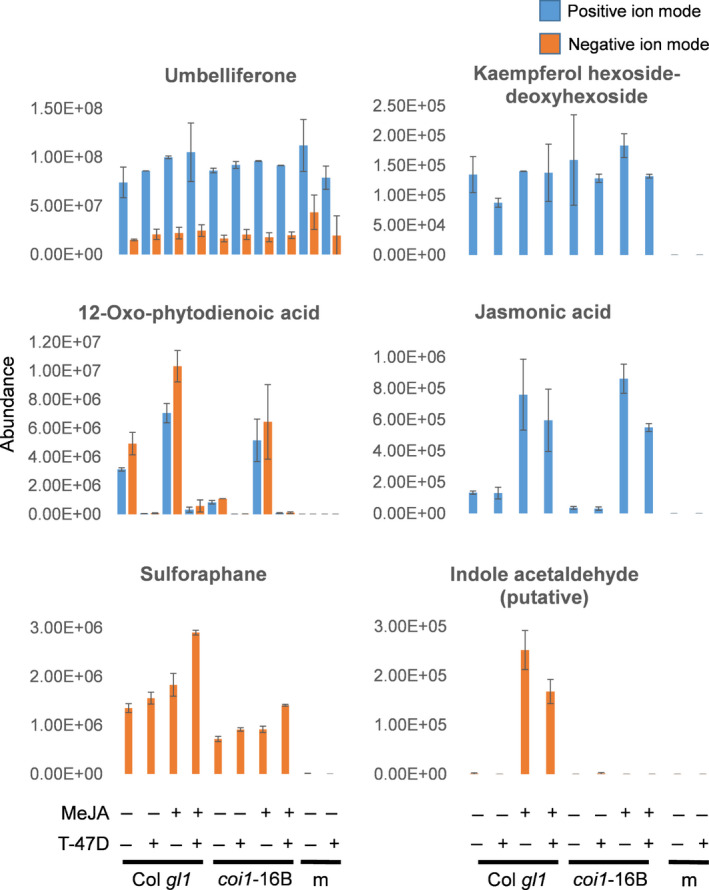
Abundance of selected Arabidopsis metabolites identified through MS/MS and database searches. Abundance data (from positive or negative ion mode) correspond to the peak areas (not normalized) determined by analysis with profinder and mass profiler professional. The compounds abundance was compared in samples containing Arabidopsis WT (wild‐type, Col Trichome differentiation protein (gl1) or mutant coi1‐16B in presence or absence of T‐47D cells and/or methyljasmonate (MeJA) against media only (m). Umbelliferone was used as an internal standard. Values denote averages ± SD (*n* = 2/3).

Accurate mass and isotope composition for the 161 plant‐specific putative features were used to calculate molecular formulas, search databases and (where present) MS/MS spectra were extracted. Several identifiable compounds were detected based on accurate mass and MS/MS spectral matches (Table S5). The abundance of these compounds was compared in samples containing Arabidopsis WT (Col *gl1*) or mutant *coi1*‐16B in the presence or absence of T‐47D cells and/or MeJA against media only (m). As expected, increased JA was detected in MeJA‐treated samples in a COI1‐independent manner (Fig. 3). JA presence was unaffected by the presence of T‐47D cells. Of the major specialized Arabidopsis metabolites, 4‐methylsulfinylbutyl isothiocyanate (sulforaphane) a breakdown product of the glucosinolate glucoraphanin (Kissen *et al*., 2009), and the flavonol glycoside kaempferol hexoside deoxyhexoside, belonging to one of the major Arabidopsis flavonoids (Veit & Pauli, 1999), were identified. Kaempferol glycoside abundance was not affected by MeJA or by the presence of T‐47D cells, and its presence was COI1‐independent. Sulforaphane concentrations were mildly induced by MeJA in Col *gl1*, albeit at lower levels in *coi‐16*B, but more strongly in the presence of T‐47D. OPDA, a biosynthetic precursor of JA (Zimmerman & Feng, 1978), was detected. OPDA accumulated in MeJA‐treated samples and at a lower concentration in *coi‐16*B, but the concentrations were dramatically reduced in presence of T‐47D cells. Of the compounds showing specific induction by MeJA only in Col *gl1*, hence COI1‐dependent, one (3 Pos, retention time 10.78 min) (Fig. 3; Tables S4, S5) allowed preliminary identification. Its abundance was decreased by the presence of T‐47D cells. Its predicted formula C10H9N0/159.0864 (measured 159.0679, 11 ppm deviation) corresponded to several candidate compounds, although it was outside the observed mass accuracy for the other identified compounds and the umbelliferone internal standard (5 ppm). In the MS/MS spectrum, 117.06 *m/z* is characteristic of indoles (e.g. indole acetaldehyde) and quinolines (e.g. 2‐methyl‐4‐hydroxyquinoline and others).

Further analysis is required to precisely identify the active ingredients in the complex plant mixture, the compounds detected in Arabidopsis through this study were potential bioactive compounds.

### Incubation with MeJA‐treated Arabidopsis altered transcripts and protein levels of cell cycle regulators in breast cancer cells

Given that MeJA (Fig. 1a) or MeJA‐treated *Col gl1* (Fig. 1c) reduced T‐47D cell numbers and that MeJA influences cell cycle progression (Fig. S1B–E), the effects on cell cycle markers were investigated.

We selected the following G1/S specific regulators (Cohen & Flescher, 2009; Caldon & Musgrove, 2010) and tested their relative gene expression and protein abundance: Cyclin‐dependent kinase 2 (CDK2, P24941), Cyclin D1 (CCND1, P24385), Cyclin D3 (CCND3, P30281), Cyclin E1 (CCNE1, P24864), cell division cycle 6 (CDC6, Q99741), proliferating cell nuclear antigen (PCNA, P12004), Cyclin‐dependent kinase inhibitor 1A (p21, Cip1, CDKN1A, P38936) and Cyclin‐dependent kinase inhibitor 1B (p27, Kip1, CDKN1B, P46527). Cell cycle progression through G1/S is mediated by Cyclin D/CDK4 or CDK6 and Cyclin E/CDK2 protein complexes and, once in S phase, CDC6 and PCNA are essential for DNA replication (Matson & Cook, 2017). However, p21 and p27 prevent Cyclin/CDK complex formation and participate in DNA damage repair (Abukhdeir & Park, 2009).

**Fig. 4:**
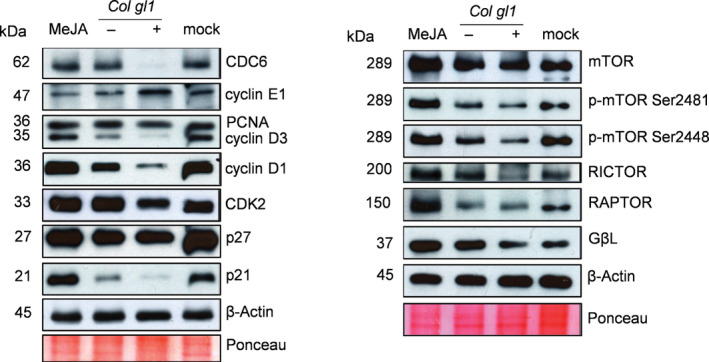
Cell cycle regulators and components of the mechanistic target of rampamycin (mTOR) signalling pathway are altered in breast cancer cells upon methyljasmonate (MeJA) treatment or incubation with Col Trichome differentiation protein (gl1) leaf disks. T‐47D cells were subjected to 2 mM methyljasmonate (MeJA) or co‐incubated with excised leaf disks of Col*gl1* seedlings treated (+) or untreated (−) with MeJA for 72 h and proteinlevels analysed. (a) Western blot detection of cell division cycle 6 (CDC6), Cyclin E1 (CYCE1), CyclinD1, CyclinD3, proliferating cell nuclear antigen (PCN), Cyclin‐dependent kinase 2 (CDK2), Cyclin‐dependent kinase inhibitor 1A (p21) and 1B (p27), in T‐47D cells. β‐Actin proteinlevels and Ponceau‐S staining were used as to determine equal loading. (b) Western blot detection of mTOR, p‐TOR Ser2481, p‐TOR Ser2448, RICTOR, RAPTOR and GβL in T‐47D cells using the indicated antibodies. Samples were harvested after 72 h treatment, along with a vehicle‐treated growth control (mock). β‐Actin proteinlevels and Ponceau‐S staining were used to determine equal loading.

The protein levels of CDC6 and CDK2 in cells in response to MeJA remained unchanged (Fig. 4a), but the gene expression levels decreased (Fig. S4). Cyclin D1, Cyclin D3, p27 and PCNA protein levels mirrored the changes in transcript levels and were unaffected by MeJA compared to levels in lysates from the mock, ethanol‐treated cells. Cyclin E1 protein levels were reduced and both p21 transcript and protein levels increased in cells treated with MeJA, compared to controls (Figs 4a, S4).

When cells were co‐incubated with leaf disks from MeJA‐treated plants, protein and transcript levels of CDC6, Cyclin D1 and CDK2 were reduced compared to cells incubated with untreated leaf disks. Cyclin D3 protein levels also were reduced, although no differences were observed in transcripts. Co‐incubation with MeJA‐treated *Col gl1* leaf disks also increased *Cyclin E1*, *p21* and *p27* transcripts, although the effects on protein levels differed; Cyclin E was increased, p21 was decreased and p27 concentrations were unaffected. PCNA protein or transcript levels were unaffected (Figs 4a, S4).

### The effect of MeJA‐treated Arabidopsis on the mechanistic target of rapamycin signalling pathway

The mechanistic target of rapamycin (mTOR) is a known therapeutic target in breast cancer (Hare & Harvey, 2017). Recent evidence suggests crosstalk between TOR and JA signalling in Arabidopsis (Song *et al*., 2017; Pérez‐Salamó *et al*., 2019). For this reason, the effects of both MeJA and MeJA‐treated Col *gl1* on mTOR in T‐47D breast cancer cells were examined. (Fig. 4b). Notably, the treatment of T‐47D cells with MeJA caused the opposite effects to those observed with the leaf disks. An increase in the protein levels of mTOR, p‐TOR (Ser2481), p‐TOR (Ser2448), RICTOR, RAPTOR and GβL was observed in MeJA‐treated cells compared to untreated controls. By contrast, incubation with MeJA‐treated Col *gl1* did not affect the protein levels of mTOR and RAPTOR, but decreased the protein abundance of phosphorylated mTORC p‐TOR (Ser2448), p‐TOR (Ser2481), RICTOR and GβL in T‐47D cells in comparison to cells incubated with the untreated leaf disks (Fig. 4b).

Overall, these data showed that both MeJA and MeJA‐treated WT Arabidopsis leaves affect mTOR protein levels. When combined with the differences in cell cycle protein data (Fig. 4a), they indicate that the component(s) involved in mediating the bioactivities on breast cancer cells are likely to be compound(s) or downstream metabolite(s) from MeJA‐treated leaf explants, distinct from those induced in T‐47D cells by direct treatment with MeJA (or indeed MeJA itself).

## Discussion

### A bioassay to assess the effect of methyljasmonate on the growth of human breast cancer cells

A systematic relationship between the production of plant specialized metabolites (Balbi & Devoto, 2008; Zhou & Memelink, 2016; Pérez‐Salamó *et al*., 2019) and the regulation of growth and development and response to stress has not yet been established. Moreover, Arabidopsis, despite having been used as a model plant for over 30 yr, has never been considered as a potential source of phytotherapeutics.

Jasmonates (JAs) elicit *de novo* transcription and translation and, ultimately, the biosynthesis of specialized metabolites in plants (Memelink *et al*., 2001). The anticancer activity of JAs has been demonstrated both *in vitro* and *in vivo* (Fingrut & Flescher, 2002; Flescher, 2005; Balbi & Devoto, 2008; Cesari *et al*., 2014; Pérez‐Salamó *et al*., 2019). However, it is not known whether this is a direct effect of JAs. Induction of specialized metabolites could underlie the increased growth inhibition of human breast cancer cells when co‐incubated with methyljasmonate (MeJA)‐treated plant samples. The effectiveness of compounds from plants also could be affected by interaction between them and the target cells. To our knowledge, there has been no systematic study comparing the effect of JAs on breast cancer and nontumourigenic breast cells.

We show that the direct cytotoxic effect of MeJA is selective for human breast cancer cells (Figs 1a, S1). Previous studies have reported low or no cytotoxicity of JAs to healthy cells compared to cancer cells (Fingrut & Flescher, 2002; Rotem *et al*., 2003; Reischer *et al*., 2007). Cytotoxicity assays (Yeruva *et al*., 2008) showed a significant decrease in the cell viability of the human breast cancer cell lines MDA‐MB‐435 and MCF‐7 at concentrations of ≥ 1.5 mM MeJA.

In our study, Arabidopsis explants treated with 50 µM MeJA had an effect on breast cancer cells (Fig. 1d) comparable to treatment with mM concentrations of MeJA. Such enhanced efficacy could be indicative of synergic effects of JAs with other compounds. The reduced sensitivity of MDA‐MB‐361 compared to T‐47D cells, could be attributable to their HER2 positivity, linked to recalcitrance to chemotherapy (Sauter *et al*., 2009). This difference emphasizes the usefulness of our bioassay to detect differences between treatments and cell types, and to detect the interactions between phytotherapeutics and cancer cells.

### Coronatine‐insensitive protein 1‐dependent jasmonic acid signalling mediates the effect of exogenous MeJA treatment of Arabidopsis explants on reducing human breast cancer cells growth

Suppression of cancer cell growth was consistently stronger when cells were co‐incubated with MeJA‐treated plant samples compared to untreated controls (Fig. 1d). The differential effect between MeJA‐treated and untreated plant samples was due to jasmonic acid (JA) signalling. This finding indicated that anticancer activity was not only a direct effect of MeJA itself, but also the result of production of JA‐regulated, Coronatine‐insensitive protein 1 (COI1)‐dependent specialized metabolites (Devoto *et al*., 2005; Pauwels *et al*., 2008; Pérez‐Salamó *et al*., 2019).

Overexpression of COI1 was found previously to affect positively the availability of metabolites such as β‐alanine, threonic acid, putrescine, glucose and myo‐inositol, thereby providing a connection between JA‐inhibited growth and stress responses (Bömer *et al*., 2018). Here, *COV99* plants overexpressing the COI1 receptor, exhibit wild‐type (WT) responses in bioassays, indicating that any observed increases in inhibition of cancer cell growth by MeJA‐treatment of Arabidopsis do not necessarily depend on the dose of the COI1 receptor.

The differential effects of MeJA treated and untreated leaf samples were consistently less pronounced in *aos* compared to the corresponding WT Col *Trichome differentiation protein* (*gl1*), indicating that the positive feedback regulatory loop and endogenous JA concentrations may contribute to the anticancer potential of MeJA‐treated Arabidopsis. In accordance with the JA‐dependency of the effects on the breast cancer cells, explants of *cev1* displayed the strongest inhibitory potential on the growth of T‐47D and MDA‐MB‐361 breast cancer cells. Higher endogenous concentrations of JAs and constitutive JA responses in the *cev1* mutant (Ellis & Turner, 2001) emphasize the role of JA‐induced specialized metabolites in breast cancer cell growth suppression. The enhanced efficacy of the MeJA‐treated samples in inhibiting the growth of breast cancer cells also could be indicative of a synergistic effect between JAs and the production of other plant‐derived compounds with anticancer activity. Notably, cell growth of the nontumourigenic MCF‐10A cells was not affected significantly following exposure to leaf disks, except for exposure to MeJA‐treated *cev1* explants. The inhibitory potential of untreated Arabidopsis leaf disks and leaf disks from MeJA‐treated plants is likely, and therefore selective, for cancer cells, demonstrating a similar selective cytotoxicity towards cancer cells as previously described for MeJA.

### Identification of Arabidopsis metabolites inhibiting breast cancer cell growth

Untargeted LC‐MS/MS identified *COI1*‐dependent and MeJA‐induced compounds in the cell media, in the presence and absence of T‐47D cells (Fig. 2; Table 1). This analysis indicated complex interactions between Arabidopsis metabolites and T‐47D cells. Two hundred and sixty‐six plant‐specific MeJA‐induced features were detected (Table S4) and some putatively identified, based on accurate mass and MS/MS spectral matches, lending confidence to our conclusion that other compounds are unknowns and awaiting discovery (Fig. 3; Table S5). The uniform abundance of the plant‐specific flavonol glycoside, kaempferol hexoside deoxyhexoside, across all samples (Veit & Pauli, 1999) validated compounds changes under varying conditions. The identification of JA in MeJA‐treated samples also validated our ability to identify possible breakdown products of MeJA and/or to detect endogenous JAs inhibiting breast cancer cell growth.

One explanation for the complex cis‐(+)‐12‐oxophytodienoic acid (OPDA) abundance pattern, induced in MeJA‐treated samples and decreased in the presence of T‐47D cells, is that MeJA inhibits endogenous JA synthesis in leaves, resulting in OPDA accumulation and secretion. Because there are four possible stereoisomers of OPDA in plants (Schaller *et al*., 1998), it is possible that that MeJA induces accumulation of a specific one. Unfortunately, the chromatography column used for LC‐MS is unable to resolve these stereoisomers. However, the T‐47D cells might metabolize OPDA (or inhibit its secretion). Our data support OPDA induction of JA‐independent specialized metabolite production (Taki *et al*., 2005) and that direct treatment with OPDA has anticancer activity by targeting Cyclin D1 (Nedret *et al*., 2008). Significantly, we showed that OPDA is produced by the plants and that it may cause a reduction in the Cyclin D1 protein levels (Fig. 4a).

Although glucosinolates, including sulforaphane, act as anticancer compounds (Mokhtari *et al*., 2018), isothiocyanates derived from glucosinolates do not play a major role in growth suppression of the human breast cancer cells (Fig. S3; Table S2).

The possible identification of a plant‐specific MeJA‐induced compound with an MS/MS spectrum characteristic of indoles and quinolines provides possible mechanistic insights into the effects of MeJA in plants. Importantly both classes of compounds have been previously identified as anticancer (Musiol, 2017) but their mechanisms of actions remained elusive.

### D‐type Cyclins, Cell division cycle 6 and Cyclin‐dependent kinase 2 are mechanistic targets of MeJA‐induced Arabidopsis bioactivities

The effect on the growth of breast cancer cells caused by MeJA‐treated plants, and by direct MeJA treatment (Figs 1, S2, S3), prompted our investigation of cell cycle markers in T‐47D cells, under both conditions, whereby strikingly differential effects were detected (Figs 4, S4). Our data suggest that JAs delay the progression of cells from G0/G1 phase into S phase inducing apoptosis (Fig. S1C,D). Stalling the cells in G0/G1 may gain time to repair cellular damage. At high doses of MeJA, irreparable damage induced cell death. Likewise, efforts to increase G2‐M arrest have been associated with enhanced apoptosis (Ehrlichová *et al*., 2005).

The action of D‐ and E‐type Cyclins, Cyclin‐dependent kinase 2 (CDK2) and the CDK inhibitor proteins p21, p27 and p57 characterize the G1 phase of the cell cycle and activation of target proteins for S‐phase progression (Caldon & Musgrove, 2010). p27 and p21 inhibit Cyclin CDK complexes in G0/G1. It has been hypothesised that PCNA downregulation induces cell cycle arrest, in association with Cyclin D1 or D3, CDK2 and p21 (Cohen & Flescher, 2009). Lack of detectable changes in expression of either p27 and PCNA in our study suggests cell type‐specific regulation of the cell cycle either by MeJA or by MeJA‐treated leaf explants. The relative stability of p27 could also be a positive indicator of better patient outcome following MeJA treatment (Alkarain *et al*., 2004).

**Fig. 5:**
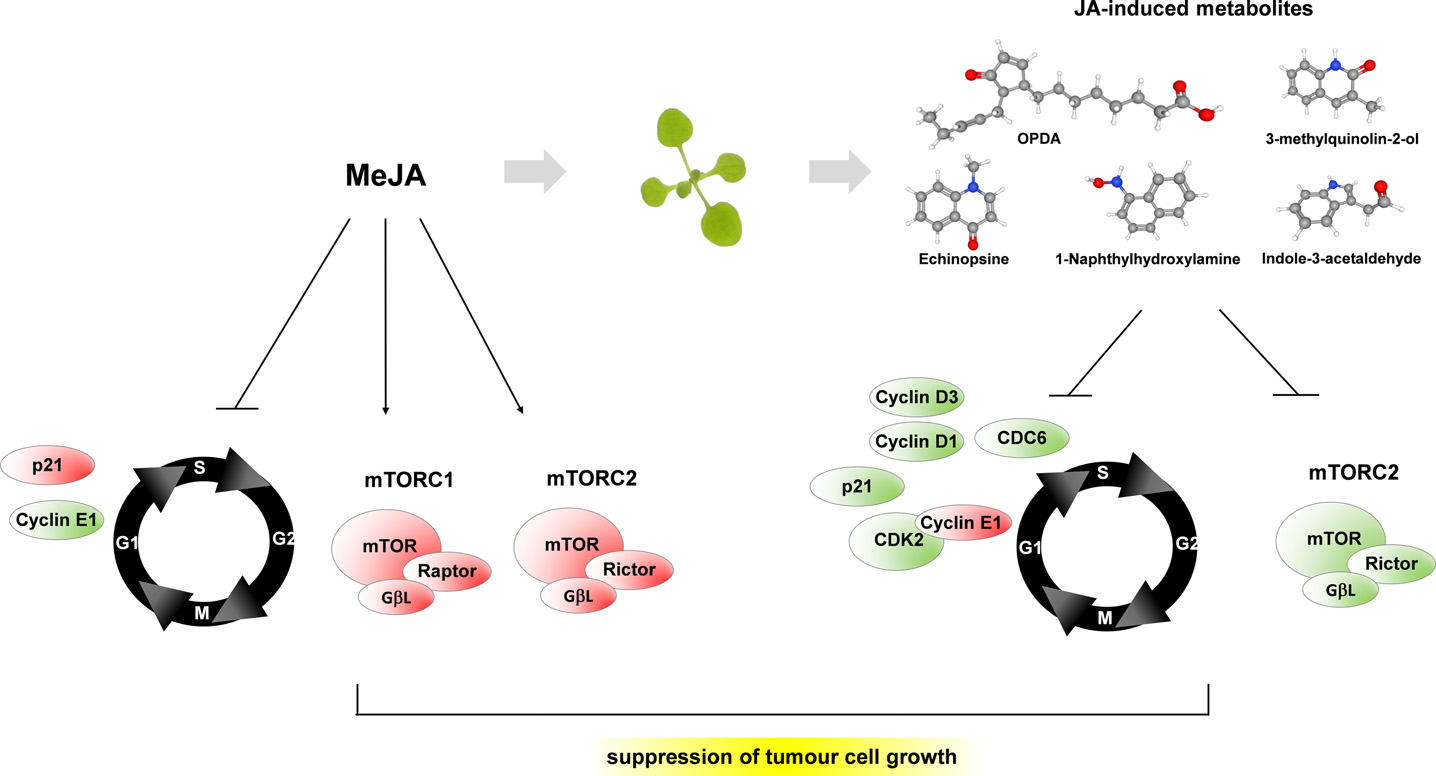
Differential regulation of breast cancer cell growth by methyljasmonate (MeJA) or MeJA‐treated leaf disks. Treatments with MeJA (left) or MeJA‐treated leaf disks (right) on breast cancer cells alter proteinlevels of different core cell cycle regulators and the mechanistic target of rampamycin (mTOR) pathway, resulting in tumour growth inhibition. MeJA treatment induces changes in Cyclin‐dependent kinase inhibitor 1A (p21) and Cyclin E1 (CYCE1)levels, while inducing the accumulation of the components of both mTORC1 and mTORC2 complexes. By contrast, incubation with MeJA‐treated leaf disks not only affects cell division cycle 6 (CDC6), Cyclin‐dependent kinase 2 (CDK2), Cyclin E1 (CYCE1), CyclinD1, CyclinD3 and Cyclin‐dependent kinase inhibitor 1A (p21), but also inhibits the accumulation of the mTORC2 complex. Names and 2D structures (PubChem) of compounds discovered through MS/MS are indicated. Red and green shapes indicate accumulation or reduction in proteinlevels, respectively.

Methyljasmonate treatment reduced Cyclin E1 (CYCE1) and increased p21 protein levels (Fig. 4a), supporting our cell cycle analysis (Fig. 4a). E‐type Cyclin activity is limiting for cells passing from G1 into S‐phase. Cyclin E binds and activates CDK2 leading to S‐phase specific gene expression (Möröy & Geisen, 2004). CDK2 also phosphorylates several components of the DNA pre‐replication complex, including cell division cycle 6 (CDC6) (Chuang *et al*., 2009). The reduction of CYCE1 and increased p21 protein levels (Figs 4a, 5), also supports the flow cytometry analysis. A different effect was observed for MeJA‐treated plants on CYCE1 and p21, highlighting the mechanistic differences between the direct effects of MeJA treatment of the breast cancer cells and incubation with MeJA‐treated plant explants.

Downregulation of CDK2 gene expression was observed following incubation with both MeJA and MeJA‐treated leaf disks (Fig. S4), although only the latter caused reduction of CDK2 protein (Figs 4a, 5). This suggests that incubation with MeJA‐treated plant explants, but not with MeJA, activate additional signalling pathways leading to CDK2 protein degradation. The association of ubiquitin‐dependent degradation of CDK2 with the arrest of tumour growth in acute myeloid leukemia (AML) cells (Ying *et al*., 2018) supports this hypothesis.

D‐type Cyclins (D1, D2 and D3) are essential for G1 phase and can limit G1/S transition (Herzinger & Reed, 1998). Our results confirmed that MeJA treatment of human breast cancer cell lines had no effect on Cyclin D1 expression at the RNA level as reported for neuroblastoma cells by Tong *et al*., (2008). Strikingly, we also showed that MeJA‐treated Arabidopsis explants substantially reduced the levels of Cyclin D proteins in human breast cancer cell lines, in accordance with studies linking the downregulation of Cyclin D1 and Cyclin D3 levels to antitumour therapy in breast cancer patients (Ortiz *et al*., 2017; Wang *et al*., 2019), hereby providing mechanistic targets for MeJA‐induced plant bioactivities inhibiting breast cancer cells growth, and further ground for these cell cycle regulators as targets of anticancer compounds.

The mammalian CDC6 is a trifunctional AAA + ATPase (Duderstadt & Berger, 2008), controlling the G1/S transition, DNA replication and cell survival (Okayama, 2012). CDC6 also controls CDK2 activity during G1/S transition and subsequent obstruction of apoptosome assembly inhibiting cell death during proliferating (Niimi *et al*., 2012). In our study, the levels of CDC6 protein were dramatically reduced by MeJA‐treated plants, supporting previous findings where CDC6 was identified as target for radiosensitivity in nasopharyngeal carcinoma (Li *et al*., 2016). CDC6 downregulation shares similarity with the effect of MeJA treatment on Arabidopsis that we demonstrated previously (Noir *et al*., 2013). JAs‐induced effects, common to both mammalian and plant cells following JA treatment, include the suppression of cell proliferation, reactive oxygen species generation, cell death induction, heat shock protein expression and mitogen‐activated protein kinase induction (Flescher, 2007; Balbi & Devoto, 2008; Cesari *et al*., 2014; Pérez‐Salamó *et al*., 2019). The list of common effects can be therefore extended to reduction in CDC6 activity, a key component in JAs‐suppressed growth in both plant and cancer cells.

### The inhibition of the mTORC2 complex is a target for MeJA‐induced plant bioactivities

In breast cancer cells, treatment with Palbociclib, a CDK4/6 inhibitor, upregulates mechanistic target of rapamycin (mTOR) whilst promoting G0/G1 cell cycle arrest (Cretella *et al*., 2018). Cell cycle arrest in the presence of active mTOR promotes senescence and geroconversion, but the inhibition of mTOR with rapamycin partially suppresses the senescent phenotype (Leontieva & Blagosklonny, 2013). In our study, different effects were caused by MeJA treatment alone and by incubation with MeJA‐treated plant explants. It is surprising that, upon MeJA treatment, mTORC1/2 protein levels increased, whereas incubation with MeJA‐treated leaf disks decreased the protein levels of the mTORC2 component Rictor (Figs 4b, 5), highlighting mechanistic differences between the two treatments. The mTORC1 complex senses nutrient status to regulate protein and lipid biosynthesis, stimulating cell growth. The mTORC2 complex also responds to growth factors, as well as regulating the actin cytoskeleton, ion transport, and cell growth and survival (Jacinto *et al*., 2004). Our findings indicate that inhibition of the mTORC2 complex is the mechanism for MeJA‐induced plant bioactivities blocking breast cancer cells growth.

In Arabidopsis, in response to positive mitogenic signals, such as light, sugar availability and hormones, the TOR signalling pathway promotes cell growth that connects to the entry and cell division cycle via multiple signalling (Henriques *et al*., 2014; Ahmad *et al*., 2019). Yet there is no evidence of crosstalk between the effects of JAs and mTOR signalling in mammalian cells. However, in plants, mTOR is known to regulate phytohormone synthesis, as well as JAs signalling (Song *et al*., 2017; Pérez‐Salamó *et al*., 2019), whereby crosstalk contributes to the trade‐off between growth and defence, by modulating JA signalling and biosynthesis regulating growth conditions (Song *et al*., 2017; Pérez‐Salamó *et al*., 2019).

Our study ascribes separate roles for MeJA and MeJA‐derived compounds from Arabidopsis impacting mTOR in the breast cancer cell cycle. It is striking that conserved crosstalk between mTOR and JAs occurs in different kingdoms in regulating the cell cycle. Further studies of these naturally occurring plant compounds will be important for improving our understanding of checkpoint modulation and potentially to develop novel clinical approaches to the treatment of human cancers.

### Conclusions

Our findings ascribe unprecedented medicinal properties to what has been considered, so far, a model plant: Arabidopsis. By studying the signalling in cancer cells, we discovered universally conserved modes of action of JAs between plant and animal cells. Overall, in our study, a synergistic effect by MeJA and by compounds induced by it on the cell cycle associates with the decreased levels of CDC6, CDK2, Cyclin D1 and Cyclin D3 (Fig. 5). Consequently, the downregulation of these cell cycle regulators could mediate the mechanism behind the reduction in breast cancer cell viability. Strikingly for future applications in cancer therapy, the action of MeJA and compounds upregulated in the Arabidopsis metabolome, target a central pivot of the highly complex mechanism controlling cell proliferation and survival. Whether the effect on cell cycle markers depends on mTOR, or on the activation of the latter by MeJA as a downstream cellular response, remains to be demonstrated.

The present study provides a new platform for the discovery of plant‐derived, bioactive compounds within complex plant mixtures while also allowing the identification of synergistic effects between phytochemicals and target cells. Most traditional chemotherapeutic agents are nonspecific but selective as they act by killing cells that divide rapidly, which is one of the main properties of most cancer cells. We validated the reproducibility of the system by undertaking assays with healthy epithelial cells; showing that MeJA‐treated Arabidopsis explants are effective in selectively modulating the proliferation of tumourigenic compared to nontumourigenic cells and discriminating between them.

The bioassay allowed production of high‐value chemicals in sufficient quantities to be detected by LC‐MS even from plants with no known medicinal pedigree, allowing untapped resources to be mined without *a priori* assumptions. The system has the potential to be adapted to identify different classes of bioactive phytochemicals. Different plants can be tested allowing direct comparison of known medicinal plants with new ones with unrecognized effects; different cell types could be used to define the specificity of bioactive phytochemicals and assays could be calibrated with the combined use of mutants or phytochemical inducers. The analysis of the metabolome within targets cells also could be performed to gain further insights into the absorption mechanisms. This study has important implications for the identificatino of metabolites with anticancer bioactivities and it will have applications in developing treatments for other diseases. Combined with recent progress in metabolic engineering and biotechnology, our approach will also facilitate production and analysis of bioactivities of valuable metabolites from plants at industrial scales.

## Author contributions

AD conceived the original idea, designed the research, performed the research, analyzed the data, wrote and edited the paper; AH and NS designed the research, performed the research, analyzed the data, wrote and edited the paper; MB and IP‐S performed the research and contributed ideas to perform it, analyzed the data, wrote and edited the paper; and AC, HVF, DS, J‐HD, PF performed the research and analyzed the data. MB and IP‐S contributed equally to this work.

## Supporting information


**Fig. S1** Methyljasmonate inhibits cell cycle progression and increases cell death.
**Fig. S2** Bright‐field microscope image of T‐47D cells after treatment with 2 mM MeJA compared to exposure of Col gl1 plant leaf disks.
**Fig. S3** Screening Arabidopsis metabolism mutants with breast cancer T‐47D and nontumourigenic MCF‐10A cells.
**Fig. S4** Transcript analysis of cell cycle marker genes in the human breast cancer cell line T‐47D upon MeJA treatment or incubation with Col gl1 leaf disks.
**Methods S1** Further details and information on plant materials, human breast cancer cell lines, treatment, antibodies and analysis.
**Notes S1** R‐Script for the analysis reported at Fig 2.

**Table S1** Human qRT‐PCR primers used in this study.
**Tables S2** Inhibition values for Arabidopsis mutants on breast cancer cells.
**Tables S3** List of 1757 FOIs across all treatments in positive and negative ion mode obtained using massprofiler.

**Table S4** Metabolite features identified.

**Table S5** Compound identification information.
Please note: Wiley Blackwell are not responsible for the content or functionality of any Supporting Information supplied by the authors. Any queries (other than missing material) should be directed to the *New Phytologist* Central Office.

## Data Availability

The data integral to the paper (fully documented LC‐MS/MS analysis) is available through https://royalholloway.figshare.com/, doi: 10.17637/rh.13079153.
