# Supplementary material for: Jasmonates induce Arabidopsis bioactivities selectively inhibiting the growth of breast cancer cells through CDC6 and mTOR
Source: New Phytol. 2020 Nov 19;229(4):2120–34. doi: 10.1111/nph.17031 (PMC8022592; doi:10.1111/nph.17031)
Supplement: Supplementary file 1 — Fig. S1 Methyljasmonate inhibits cell cycle progression and increases cell death. Fig. S2 Bright‐field microscope image of T‐47D cells after treatment with 2 mM MeJA compared to exposure of Col gl1 plant leaf disks. Fig. S3 Screening Arabidopsis metabolism mutants with breast cancer T‐47D and nontumourigenic MCF‐10A cells. Fig. S4 Transcript analysis of cell cycle marker genes in the human breast cancer cell line T‐47D upon MeJA treatment or incubation with Col gl1 leaf disks. Methods S1 Further details and information on plant materials, human breast cancer cell lines, treatment, antibodies and analysis. Notes S1 R‐Script for the analysis reported at Fig 2. [file NPH-229-2120-s004.pdf]

***New Phytologist* Supporting Information**

Title: Jasmonates induce Arabidopsis bioactivities selectively inhibiting the growth of breast cancer cells through CDC6 and mTOR

Authors: Moritz Bömer, Imma Pérez-Salamó, Hannah V. Florance, Deborah Salmon, Jan-Hendrik Dudenhoffer, Paul Finch, Aycan Cinar, Nicholas Smirnoff, Amanda Harvey and Alessandra Devoto

Article acceptance date: 26 September 2020

The following Supporting Information is available for this article:

Supplemental Figures (1-4)

Fig.S1. Methyl jasmonate inhibits cell cycle progression and increases cell death.
Fig.S2. Brightfield microscope image of T-47D cells after treatment with 2 mM MeJA compared to exposure of Colgl1 plant leaf disks.
Fig.S3. Screening Arabidopsis metabolism mutants with breast cancer T-47D and non-tumorigenic MCF-10A cells
Fig.S4. Transcript analysis of cell cycle marker genes in the human breast cancer cell line T-47D upon MeJA treatment or incubation with Col gl1 leaf disks

Supplemental figure legends

Supplemental Tables (I-V)

Table S1 Human QRT-PCR primers used in this study. Excel file
Table S2 Inhibition values for Arabidopsis mutants on breast cancer cells. Excel file
Table S3 List of 1757 features of interest (FOIs) across all treatments in positive and negative ion mode obtained using MassProfiler. Excel file
Table S4 Metabolite features identified. Excel file
Table S5 Compound identification information. Excel file

Notes S1 - R-Script for the analysis reported at Fig 2

R-script used for comparative metabolite analysis to identify Features of Interest (FOIs)

Supporting Information Methods S1

Further details and information on plant materials, human breast cancer cell lines, treatment, antibodies and analysis

References to Methods S1

Fig. S1 Methyl jasmonate inhibits cell cycle progression and increases cell death in breast cancer cells.

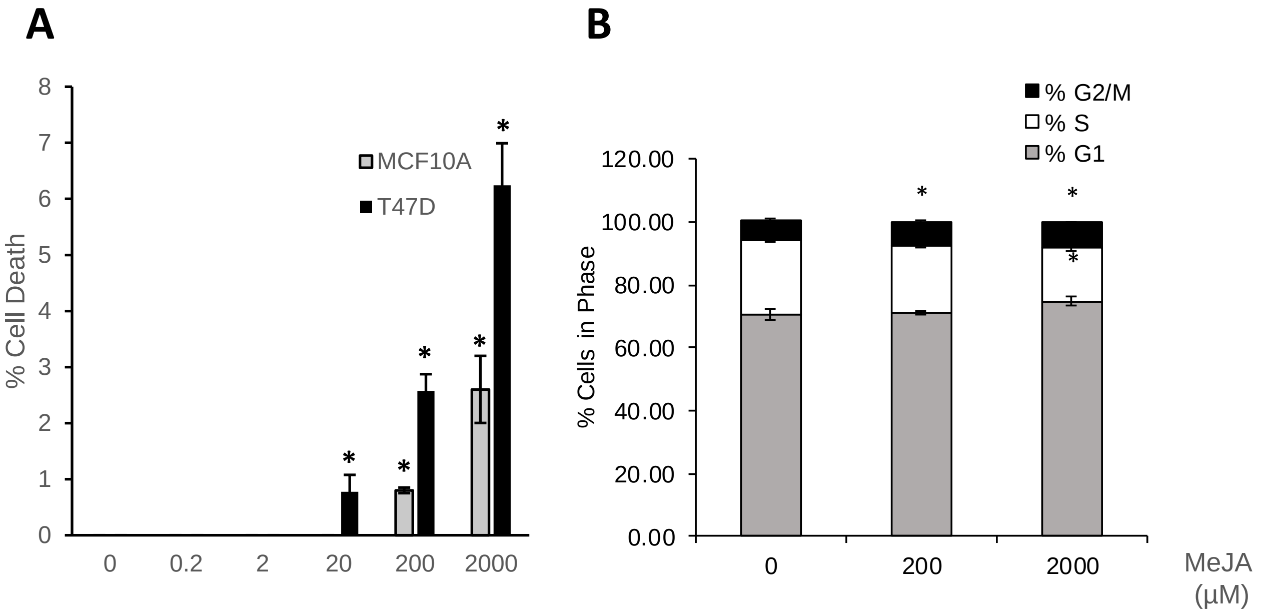

C

Flow cytometry analysis of cell cycle phases in T47D cells

	MeJA (µM)	G1	S-phase	G2/M
T47D (%)	0	70.45%	23.84%	6.05%
	200	71.39%	20.96% (P<0.003)	7.61% (P<0.05)
	2000	74.66% (P<0.05)	17.33% (P<0.003)	7.84% (P<0.008)

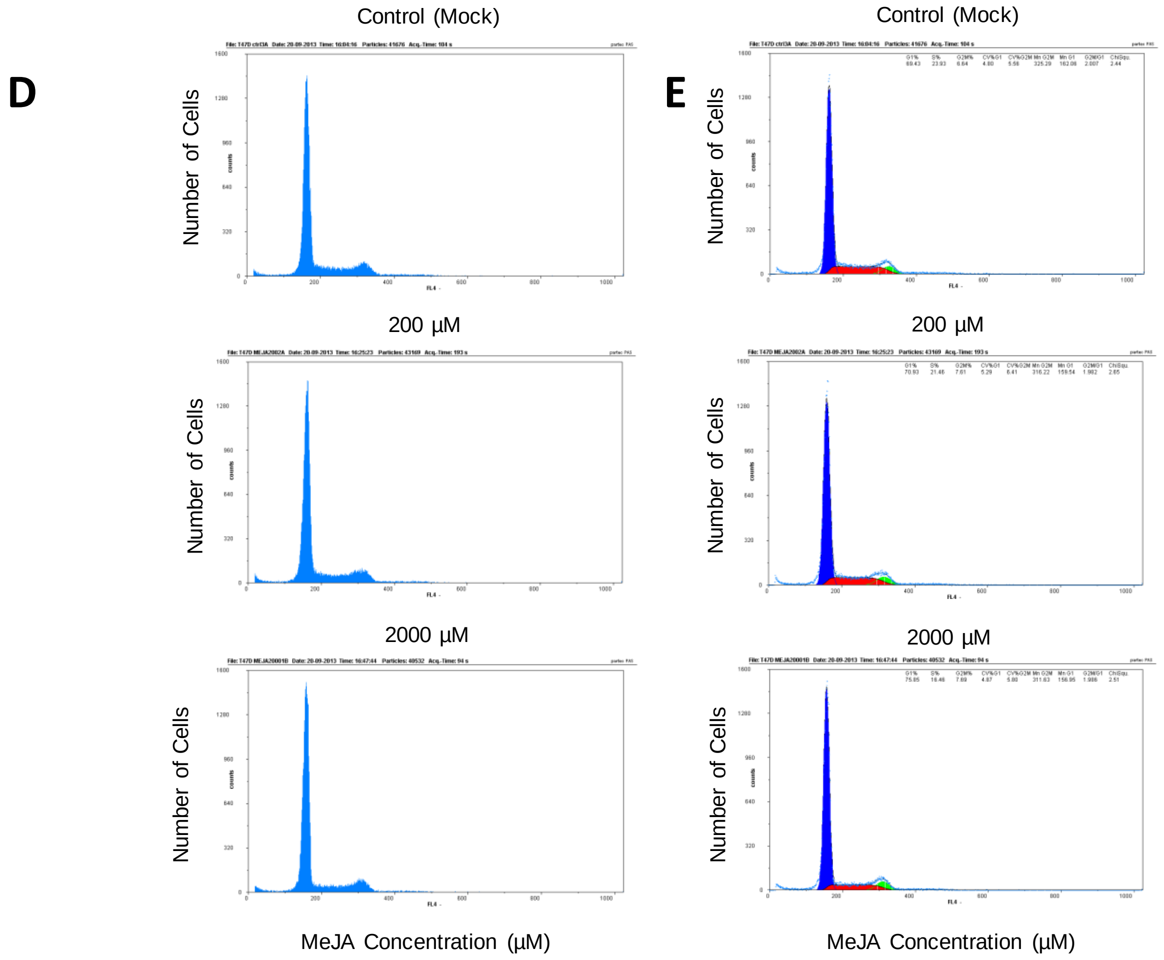

Fig. S1 Methyl jasmonate inhibits cell cycle progression and increases cell death in breast cancer cells. Cells were treated with MeJA at the indicated concentrations for 72 hours and analysed for cell death by trypan blue assay (A) or cell cycle phase by flow cytometry (B, C, D).

Increasing concentrations of MeJA affect cell death, in both T-47D and MCF10A cells with the tumour cell line being more sensitive at 20µM (A) . MeJA treatment for 72 hours resulted in an increase in the percentage of T-47D cells in G2/M with a decrease in S-phase at both 200µM and 2000µM. At 2000µM, there was also an increase in the proportion of cells in G0/G1. Significance was evaluated against ethanol treated cells. $^*P<0.05$ (B, C). Values denote averages +/- SD.

Flow cytometry peaks (D) and cell cycle analysis (E) showing the effects of MeJA on cell cycle progression observed through flow cytometry.

Fig. S2 Brightfield microscope image of the human breast cancer cell line T-47D cells after treatment with 2 mM MeJA compared to exposure of Colgl1 plant leaf disks.

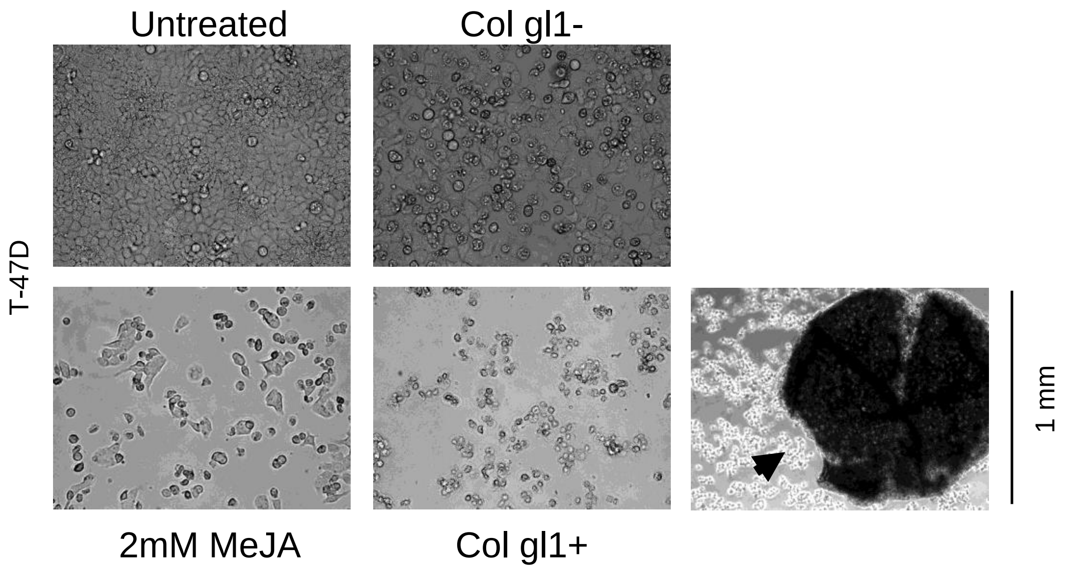

Fig. S2 Brightfield microscope image of the human breast cancer cell line T-47D cells after treatment with 2 mM MeJA compared to exposure of Colgl1 plant leaf disks. Bioassay conditions were scaled up accordingly applying 36 leaf disks to 1.2ml of T-47D cells. T-47D cells have been grown in 12 well plates (1.2 ml) and were seeded at 15.000 per 100 µl. Both treatments induce growth arrest and morphologic changes typically observed upon cell death, in particular for the MeJA treated plant leaf disks. Arrow indicates leaf disk of 1 mm diameter for size comparison. Cells were photographed through brightfield microscope under 100 x magnifications, scale bar 1 mm. T-47D breast cancer cells were treated with 2 mM MeJA or exposed to plant leaf disks of Arabidopsis wild-type background (11 DAS old) treated (+) or untreated with 50 µM MeJA for 72 hours and pictures were taken.

Fig. S3 Screening Arabidopsis metabolism mutants with breast cancer T-47D and non-tumorigenic MCF-10A cells

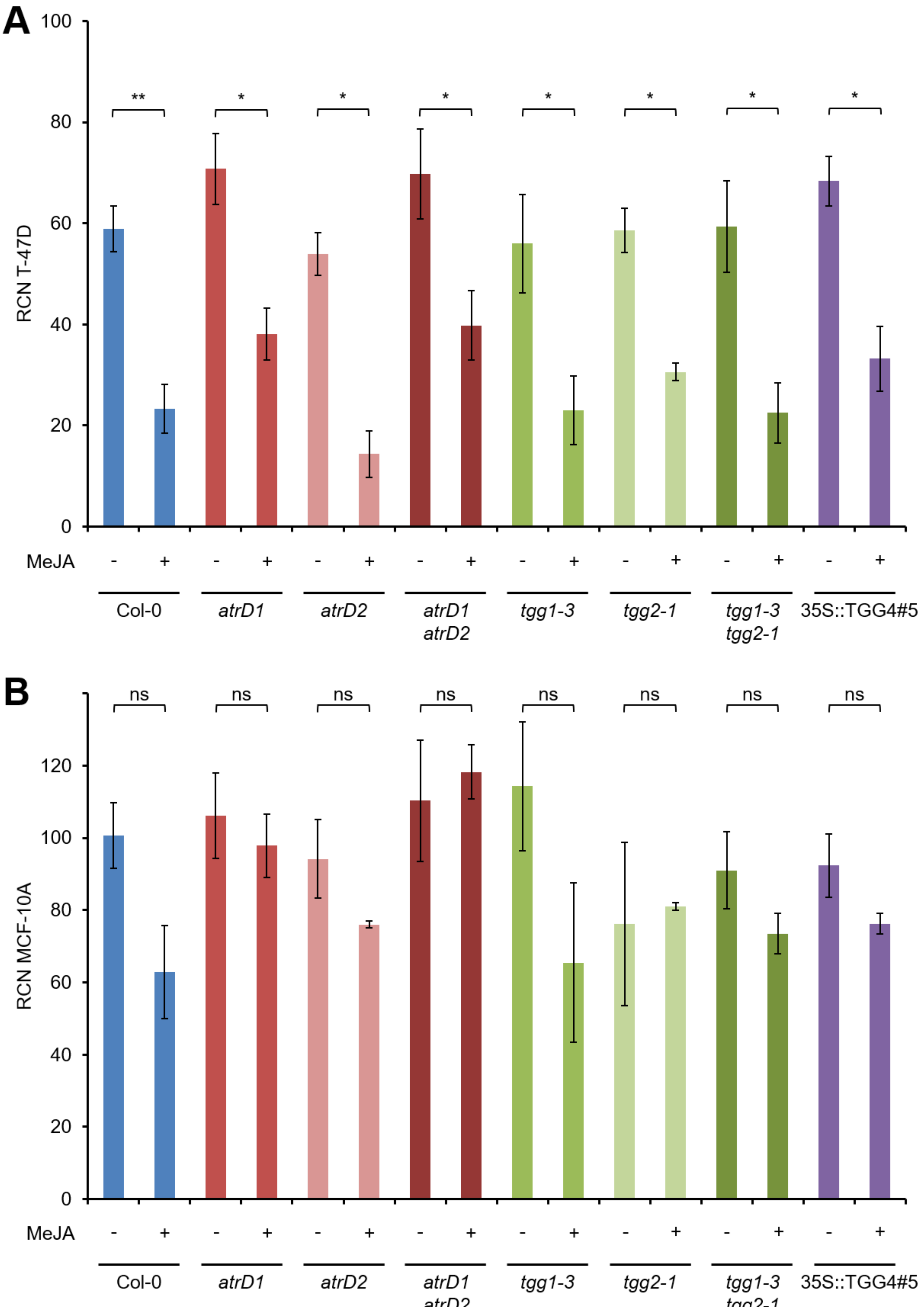

Fig. S3 Screening Arabidopsis metabolism mutants with breast cancer T-47D and non-tumorigenic MCF-10A cells

The Arabidopsis myrosinase *tgg* mutants (Barth and Jander, 2006) were tested to determine whether isothiocyanates derived from glucosinolates play any role in growth suppression of human breast cancer cells. The *atr* mutations contribute to the upregulation of P450 enzymes for indole glucosinolate synthesis (Smolen and Bender, 2002). The *tgg* mutants including the segregating over-expression line *35S::TGG4#5* (Bednarek et al., 2009) as well as the *atr* tryptophan pathway regulation mutants (Bender and Fink, 1998; Smolen et al., 2002) were assessed for their impact on T-47D cells.

Inhibition of cell growth in T-47D (A) and MCF-10A (B) human breast cancer cells upon co-incubation with excised leaf disks of WT background Col-0, homozygous T-DNA insertion lines *tgg1-3*, *tgg2-1*, homozygous double knock out *tgg1-3 tgg2-1*, the over-expression line *35S::TGG4#5*, dominant *atr* mutants *atrD1*, *atrD2*, and double mutant *atrD1 atrD2* grown for 11 DAS on media plates and treated (+) or untreated (-) with 50 µM MeJA for 24h. The data are presented as RCN in % compared to the growth control, where coloured bars correspond to the mean of three to four independent experiments with 4 technical replicates each (error bars denote standard error of the mean). *$P<0.05$; **$P<0.01$. For detailed P- values see Table S1.

Fig. S4 Transcript analysis of cell cycle marker genes in the human breast cancer cell line T-47D upon MeJA treatment or incubation with Col gl1 leaf disks

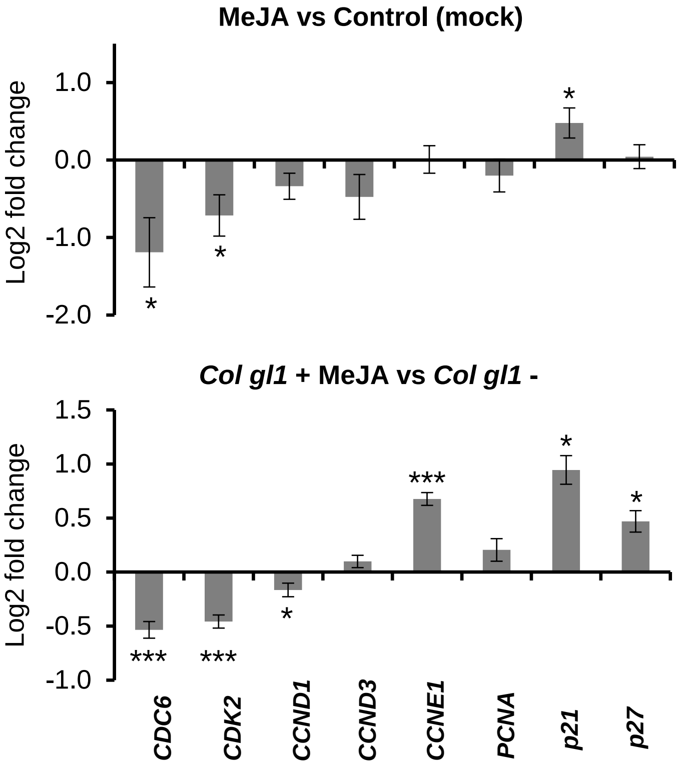

Fig. S4 Transcript analysis of cell cycle marker genes in the human breast cancer cell line T-47D upon MeJA treatment or incubation with Col gl1 leaf disks

T-47D cells were subjected to 2 mM MeJA or co-incubated with excised leaf disks of Col *gl1* seedlings treated (+) or untreated (-) with MeJA for 72 hours and gene expression and levels analysed. Gene expression profiles (log2) of *CDK2, CDC6, CYCLIN D1, D3, E1, PCNA, P21, P57, P27* and *P12*. *β-ACTIN* gene expression was used as reference gene. Data are represented as mean of n=5-6 independent experiments ± SEM. Asterisks indicate significance scores between the mock (vehicle treatment) and 2 mM MeJA treatment and between cells exposed to Col *gl1* leaf disks treated or untreated with MeJA. (**P*<0.05; ***P*<0.01; ****P*<0.001). For detailed P- values see Table S1.

Notes S1 - R-Script for the analysis reported at Fig 2
R-script used for comparative metabolite analysis to identify Features of Interest (FOIs)

```
### using additional packages ###
library(vegan)
library(pheatmap)
library(dendextend)
library(magrittr)
library(multcomp)
library(svMisc)
##################################

### own function definitions ###

# map() maps each value within a given vector onto its corresponding value in a new vector with
custom dimension #
map <- function(x, from=0, to=1)
{
  (x - min(x, na.rm = T)) / max(x - min(x, na.rm = T), na.rm = T) * (to - from) + from
}
# shift shifts a vector by n positions to create a its lagged version #
shift<-function(x, n, append=F)
{
  if(n==0)
  {
    stop('x must be non-zero integer')
  }
  if(n>0)
  {
    s<-n
    x1<-x[-c(0:s)]
    if(append==F)
    {
      x2<-c(x1, rep(NA, n))
    }
    if(append==T)
    {
      x2<-c(x1, x[c(0:s)])
    }
  }
  if(n<0)
  {
    s<-length(x)+n+1
    x1<-x[-c(s:length(x))]
    if(append==F)
    {
      x2<-c(rep(NA, abs(n)), x1)
    }
    if(append==T)
    {
      x2<-c(x[c(s:length(x))], x1)
    }
  }
  return(x2)
}
'%!in%' <- function(x,y)!('%in%'(x,y))
```

```
##########################################################################################################################
bcc <- read.delim("C:/Users/Jan/Documents/bcc_raw.txt", header=T)


bcc.mtx<-as.matrix(decostand(bcc[, -c(1:5)],    "standardize", margin=2))

compound<-colnames(bcc[, -c(1:5)])
colnames(bcc.mtx)<-compound




#sd(bcc.mtx[,100])
#mean(bcc.mtx[,100])

rownames(bcc.mtx)<-bcc$sample


col.hc <- hclust(vegdist(bcc.mtx, "euclidean"), "ward.D2")
row.hc <- hclust(vegdist(t(bcc.mtx), "euclidean"), "ward.D2")



############# check color gradient ########
colv <- colorRampPalette(c("blue",
                          "lightgrey",
                          "red"))(n = 100)
#brks <- quantile(bcc.mtx, (c=seq(0,1,length.out = 10)))
brks<-seq(min(bcc.mtx),max(bcc.mtx), length.out = 100)

plot(0, type="n", axes=T, xlim=c(min(brks), max(brks)), ylim=c(0,1), ann=F, xaxs="i", yaxs="i")
rect(shift(brks,1),0,brks,1, border = colv, col = colv)
lines(density(bcc.mtx)$x, map(density(bcc.mtx)$y), lwd=2, col="black")



ann.column<-data.frame(MeJA = bcc$meja,
                       WT = bcc$wt,
                       BCC = bcc$bcc
)

row.names(ann.column)<-bcc$sample


ann.row<-data.frame(A1 = ifelse(compound %in% A1.name, "T", "F"),
                    A2 = ifelse(compound %in% A2.name, "T", "F"),
                    B1 = ifelse(compound %in% B1.name, "T", "F"),
                    B2 = ifelse(compound %in% B2.name, "T", "F"),
                    C1 = ifelse(compound %in% C1.name, "T", "F"),
                    C2 = ifelse(compound %in% C2.name, "T", "F"))

row.names(ann.row)<-compound
```

```
ann.colors <- list(
  WT = c("wt1" = "cadetblue4",
        "wt0" = "cadetblue3"),

  MeJA = c("meja1" = "darkseagreen4",
          "meja0" = "darkseagreen3"),

  BCC = c("bcc1" = "coral4",
         "bcc0" = "coral3"),

  A1 = c("T" = "red",
        "F" = "lightgrey"),

  A2 = c("T" = "red",
        "F" = "lightgrey"),

  B1 = c("T" = "red",
        "F" = "lightgrey"),

  B2 = c("T" = "red",
        "F" = "lightgrey"),

  C1 = c("T" = "red",
        "F" = "lightgrey"),

  C2 = c("T" = "red",
        "F" = "lightgrey")

)



# o.new<-c(1,2,3,4,
#          8,7,6,5,
#          12,11,10,9,
#          13,14,15,16)

o.new<-c(13,14,15,16,
        11,12,9,10,
        7,8,5,6,
        1,2,3,4)



hm<-pheatmap(t(bcc.mtx),
        color = colv,
        breaks=brks,
        #cluster_cols = col.hc,
        cluster_cols = rotate(col.hc, o.new),
        cluster_rows = row.hc,
        annotation_col = ann.column,
        annotation_row = ann.row,
        annotation_colors = ann.colors,
        show_rownames = T,
        show_colnames = F,
        annotation_names_row = T,
```

```
	#labels_col = labels.col,
	fontsize_row = 0.5
	#main = "not standardized"
	#display_numbers = T,
	#fontsize_number = 0.5
)


dev.off()



hm

points(1,1)




bcc.env<-bcc[, c(1:5)]
bcc.env$trt<- with(bcc, paste(wt, meja, bcc, sep="_"))



### NMDS ###
# bcc.mds<- metaMDS(bcc.mtx, distance = "euclidean", k=2, trymax=50)
# plot(bcc.mds, type="n",axes=T, display = c("sites"), choices=c(2,1), cex.axis=0.8, xlab=NA,
ylab=NA)
# points(bcc.mds, display = c("sites"), cex=1, pch=19, col="blue")
# text(bcc.ord, display = c("sites"), labels = plant.v, col = "blue")

dist(bcc.mtx)
### PCA ###
bcc.pca<-rda(bcc.mtx)
plot(bcc.pca, type="n",axes=T, display = c("sites"), choices=c(2,1), cex.axis=0.8, xlab=NA,
ylab=NA)
points(bcc.pca, display = c("sites"), cex=1, pch=19, col=ifelse(bcc.env$bcc=="bcc0","blue", "red"))
#ordispider(bcc.pca, groups = bcc.env$wt)
ordihull(bcc.pca, groups = bcc.env$bcc)
ordihull(bcc.pca, groups = bcc.env$wt)

ordispider(bcc.pca, groups = bcc.env$bcc, label = T, col=c("coral3", "coral4"), lwd=3, border = F, fill
= c("coral3", "coral4"))
ordispider(bcc.pca, groups = bcc.env$wt, label = T, col=c("cadetblue3", "cadetblue4"), lwd=3)
ordispider(bcc.pca, groups = bcc.env$meja, label = T, col=c("darkseagreen3", "darkseagreen4"),
lwd=3)

ordiellipse(bcc.pca, groups = bcc.env$bcc, kind = "ehull", col=c("blue", "red"))

ordiellipse(bcc.pca, groups = bcc.env$bcc, kind = "ehull", col=c("blue", "red"))
ordiellipse(bcc.pca, groups = bcc.env$bcc, draw='polygon', alpha=0.2, border=F, col=c("blue",
"red"))

ordicluster(bcc.pca, col.hc)
#ordipointlabel(bcc.pca, display = c("sites"), cex=1, pch=19, col="blue")

summary(bcc.pca)
```

```
#####################################################

trt<- with(bcc, paste(wt, meja, bcc, sep="_"))

# m<-lm(bcc.mtx[,100]~0+trt)
# summary(m)

K<-rbind(
  "wt1_meja1_bcc1 > wt1_meja0_bcc1" =   c(0, 0, 0, 0 ,0 ,-1, 0, 1), # A1
  "wt1_meja1_bcc1 > wt0_meja1_bcc1" =   c(0, 0, 0, -1 ,0 ,0, 0, 1), # B1
  "wt1_meja0_bcc1 > wt0_meja0_bcc1" =   c(0, -1, 0, 0 ,0 ,1, 0, 0), # C1

  "wt1_meja1_bcc0 > wt1_meja0_bcc0" =   c(0, 0, 0, 0 ,-1 ,0, 1, 0), #A2
  "wt1_meja1_bcc0 > wt0_meja1_bcc0" =   c(0, 0, -1, 0 ,0 ,0, 1, 0), #B2
  "wt1_meja0_bcc0 > wt0_meja0_bcc0" =   c(-1, 0, 0, 0 ,1 ,0, 0, 0)  #C2
)



A1.name<-NULL
A1.coef<-NULL
A2.name<-NULL
A2.coef<-NULL
B1.name<-NULL
B1.coef<-NULL
B2.name<-NULL
B2.coef<-NULL
C1.name<-NULL
C1.coef<-NULL
C2.name<-NULL
C2.coef<-NULL




N<-length(colnames(bcc.mtx))
P<-0.05

for(i in 1:N)
{
  lm(bcc.mtx[,i]~0+trt)%>%
    glht(linfct = K, alternative = "two.sided")%>%
    summary() -> sg
  if(sg$test$pvalues[1]<=P && sg$test$coefficients[1] > 0)
  {
    A1.name<-append(A1.name, colnames(bcc.mtx)[i])
    A1.coef<-append(A1.coef, sg$test$coefficients[1])
  }
  if(sg$test$pvalues[2]<=P  && sg$test$coefficients[2] > 0)
  {
    B1.name<-append(B1.name, colnames(bcc.mtx)[i])
    B1.coef<-append(B1.coef, sg$test$coefficients[2])
  }
  if(sg$test$pvalues[3]>=P)
  {
```

```
    C1.name<-append(C1.name, colnames(bcc.mtx)[i])
    C1.coef<-append(C1.coef, sg$test$coefficients[3])
  }
  if(sg$test$pvalues[4]<=P  && sg$test$coefficients[4] > 0)
  {
    A2.name<-append(A2.name, colnames(bcc.mtx)[i])
    A2.coef<-append(A2.coef, sg$test$coefficients[4])
  }
  if(sg$test$pvalues[5]<=P  && sg$test$coefficients[5] > 0)
  {
    B2.name<-append(B2.name, colnames(bcc.mtx)[i])
    B2.coef<-append(B2.coef, sg$test$coefficients[5])
  }
  if(sg$test$pvalues[6]>=P)
  {
    C2.name<-append(C2.name, colnames(bcc.mtx)[i])
    C2.coef<-append(C2.coef, sg$test$coefficients[6])
  }
  progress(i, max.value = N)
}


length(A1.name) # "wt1_meja1_bcc1 > wt1_meja0_bcc1"
length(A2.name) # "wt1_meja1_bcc0 > wt1_meja0_bcc0"
length(B1.name) # "wt1_meja1_bcc1 > wt0_meja1_bcc1"
length(B2.name) # "wt1_meja1_bcc0 > wt0_meja1_bcc0"
length(C1.name) # "wt1_meja0_bcc1 > wt0_meja0_bcc1"
length(C2.name) # "wt1_meja0_bcc0 > wt0_meja0_bcc0"




writeClipboard(as.character(A1.name))
writeClipboard(as.character(A1.coef))
writeClipboard(as.character(A2.name))
writeClipboard(as.character(A2.coef))
writeClipboard(as.character(B1.name))
writeClipboard(as.character(B1.coef))
writeClipboard(as.character(B2.name))
writeClipboard(as.character(B2.coef))
writeClipboard(as.character(C1.name))
writeClipboard(as.character(C1.coef))
writeClipboard(as.character(C2.name))
writeClipboard(as.character(C2.coef))

compound
writeClipboard(as.character(compound))



library(venn)

length(A1.name[A1.name %in% A2.name])

v.bcc1<-list(A1 = A1.name,
       B1 = B1.name,
       C1 = C1.name)
```

```
v1<-venn(v.bcc1, zcolor = "style")
v1
I1<-attr(v1, "intersections")[[7]]


I1

venn(list(A1.name, A2.name, B1.name, B2.name, C1.name, C2.name),
    zcolor = "style")
```

Methods S1

Jasmonates induce Arabidopsis bioactivities selectively inhibiting the growth of breast cancer cells through CDC6 and mTOR

Plant materials and treatment

The Arabidopsis lines *coi1*-16 (At2g39940; Ellis and Turner, 2002), cleaned from the *pen2* mutation by backcross (*coi1*-16B; Westphal et al., 2008; Noir et al 2013), *aos* (At5g42650; N6149), *cev1* (At5g05170; Ellis and Turner, 2001), *COV*99 transgenic *A. thaliana* T2 line expressing COI1 as haemagglutinin (HA) C-terminal fusion proteins (COI1::HiA; Devoto et al., 2002), their genetic background Col *gl1* (or Col5, N1644), the myrosinase mutants *tgg1*-3 and *tgg2*-1 and the double mutant *tgg1*-3 *tgg2*-1 (Barth and Jander, 2006), the segregating over-expression line 35S::TGG4#5 (Bednarek et al., 2009), the tryptophan pathway mutants *atr1D, atr2d* (Bender and Fink, 1998) and the double mutant *atr1D atr2d* (Smolen et al., 2002), and their genetic background Arabidopsis ecotype Columbia (Col-0, N1093) were used. *A.thaliana* seeds were grown, and plant material treated with MeJA as described previously (Noir et al., 2013).

Seeds germinating on solid media were grown vertically so as to transfer seedlings of 10 DAS age to MeJA containing media without damaging the root network and thus avoiding wounding responses. Untreated plant samples nevertheless were transferred to media plates at the same time-point to exclude any effect induced by transfer stress.

Jasmonic acid ((1R,2R)-3-Oxo-2-(2Z)-2-pentenyl-cyclopentylethanoic acid) and methyl-jasmonate (methyl {(1R,2R)-3-oxo-2-[(2Z)-2-pentenyl-cyclopentyl}acetate) were purchased from Sigma (Poole, UK) and Bedoukian Research (U.S) respectively.

Human breast cancer cell lines

T-47D, MDA-MB-361 and MCF-10A cells were originally purchased from the American Type Culture Collection (ATCC; http://www.lgcstandards-atcc.org/). MCF-10A cells were routinely

maintained in 1:1 Dulbecco's modified Eagle's medium (DMEM)/F12 supplemented with 10% foetal bovine serum, epidermal growth factor (EGF; 20 ng/ml), insulin (5 µg/ml), hydrocortisone (1 µg/ml), 2 mM glutamine and penicillin/streptomycin (10 U/ml penicillin, 10 µg/ml streptomycin). MDA-MB-361 and T-47D cells were maintained in RPMI 1640 supplemented with 10% foetal bovine serum and penicillin/streptomycin. All cultures were maintained at 37°C in a humidified atmosphere with 5% CO_2 in air.

Plant leaf disk bioassay using human breast cancer cells

The antiproliferative effect of Arabidopsis plants aged 11 days after sowing (DAS) +/- 50 µM MeJA for 24h was evaluated using the MTT cell viability assay. Seeds germinating on solid media were grown vertically so as to transfer seedlings of 10 DAS age to MeJA containing media without damaging the root network and thus avoiding wounding responses. Untreated plant samples nevertheless were transferred to media plates at the same time-point to exclude any effect induced by transfer stress.

Relative quantification of the cell proliferation of the human breast cancer cell lines T-47D and MDA-MB-361 and the non-tumourigenic cell line MCF-10A was assessed by MTT assay and 96 well plate readings (at 562nm with a reference wavelength of 630nm) were presented as relative cell number (RCN) of viable cells expressed as a percentage (%) compared to the growth control. To further illustrate the differential effect between the treatments the bioassay data were presented as % of inhibition of the cell growth. Here we relate the RCN of treated plant samples to the RCN of the untreated control samples using the formula (100-((100/RCN untreated)*RCN treated)). A positive inhibition value means that MeJA-treated plant samples inhibited the growth of the human breast cancer cells more strongly than the untreated control samples.

MTT cell viability assay

Cells were seeded in 96-well plates at 15,000 cells/well in 100µl medium. After 24 hours cells were treated with ethanol, MeJA (over a range of concentrations between 0.2 and 2000µM)

or excised leaf disks and incubated for a further 72 hours at 37ºC. After 72h of co-incubation, cell viability assay (3-[4,5-dimethylthiazol-2-yl]-2,5-diphenyl tetrazolium bromide (MTT) assay) was performed using the MTT Reagent A (Chemicon (Millipore); cat. No. CT01-5; Mosmann, 1983; Green et al., 1984). MTT assay was carried out according to the manufacturer's instructions with 2-3h of incubation time with the MTT solution.

Trypan blue cell viability assay

Cytotoxic effects of MeJA on the cells were evaluated using a trypan blue inclusion assay. The cells were seeded in 24-well plates in a range of 50,000 cells/well in a final volume of 1 ml of medium and were allowed to attach overnight. After 24h the medium was refreshed, the cells were treated with JA or MeJA for 72h over a range of concentrations (0-2000 µM). At the end of incubation, the cells were harvested, resuspended in medium and mixed with an equal volume of 0.2% trypan blue. Viable, trypan excluding, and non-viable, trypan including, (blue) cells were counted with using an improved Neubauer haemocytometer and the percentage of non-viable (dead) cells determined.

Western blotting

Cells were seeded at $2.5x10^4$ cells/cm^2, allowed to adhere overnight and then treated with 2 mM MeJA, equal volumes of ethanol (mock) or co-incubated with excised leaf disks. After 72h incubation the cells were detached, centrifuged and the cell pellet was washed twice with PBS. The cell cycle antibodies were purchased from Abcam (Cyclin E1 (P24864, rabbit mAb ab33911), Cyclin D3 (P30281, rabbit mAb ab52598)) or Cell Signaling Technology (CyclinD1 (P24385, rabbit mAb #2978) CDK2 (P24941, rabbit mAb #2546), p21 (P38936, rabbit mAb #2947), p27 (P46527, rabbit mAb #3688) , PCNA (P12004, rabbit mAb #13110), CDC6 (Q99741, rabbit mAb #3387) and the mTOR pathway (mTOR Pathway Antibody Sampler Kit #9964). . β-Actin was used as loading control (P60709, rabbit mAb #5125, CST).).

Cell cycle analysis

As described in the western blotting assay the same number of cells were treated with 200 µM JA or MeJA and incubated for 72h prior to harvesting. Subsequently, the cells were fixed with 4% paraformaldehyde.

Quantitative RT-PCR (qRT-PCR)

Purification of total RNA derived from human breast cancer cells was extracted using the NucleoSpin RNA/Protein Kit (Macherey-Nagel). In order to gain enough RNA yields for reverse transcription, the bioassay needed to be scaled up to 2.7 ml volumes of T-47D cells (seeded in 6 well plates) and a total of 81 leaf disks per well. MTT assay was performed alongside using the typical bioassay parameters.

Standard amplification protocols were used and carried out on a Rotor-gene Q real-time PCR machine (Qiagen, USA). All reactions took place in triplicate. Melting curves were analysed to check primer specificity. Transcript levels were normalised against the reference gene β-Actin (NM_001101.4) and quantified using the $\Delta\Delta C_T$ calculation. All primers were designed using Primer 3 tool (Massachusetts Institute of Technology, Massachusetts) using the default settings and synthesised by Sigma-Aldrich (Poole, Dorset, UK).

Metabolite profiling by liquid chromatography-mass spectrometry (LC-MS/MS)

Human breast cancer cells, co-incubated with excised leaf disks from *A. thaliana* using the experimental setup of the bioassay described, and combining 16 wells (100 µl volume per well) to a total volume of 1.6 ml, were removed by centrifugation (15.000 g, 5 min) and the supernatant was snap frozen in liquid nitrogen.

Samples were thawed on ice and 300 µl of each was extracted for 60 min at -20 ºC in 900 µl acetonitrile (ACN) containing 1% acetic acid and 0.6 µl umbelliferone (3.6 mg/ml stock), used as the internal standard for sample recovery quality control. After centrifugation (15 min at 15.000 g, 4 ºC), the supernatant was transferred to a fresh tube and dried using a vacuum concentrator (SpeedVac, ThermoFisher Scientific) at 4 ºC for ~2h. The dry pellet was subsequently reconstituted in 150 µl of 80% methanol, mixed and centrifuged (15 min at

15.000 g, 4 ºC). The supernatant was filtered through a 0.2 µm PVDF syringe filter (Chromacol, ThermoFisher Scientific) into a glass autosampler vial.

Metabolite profiling was performed using a QToF (Quadrupole Time of Flight) 6520 mass spectrometer (Agilent Technologies, Palo Alto, CA, USA) coupled to a 1200 series Rapid Resolution HPLC system. Five microlitres of sample extract was loaded onto a Zorbax StableBond C18 1.8 µm (particle size), 2.1 x 100 mm (diameter by length) reverse-phase analytical column (Agilent Technologies). For positive ion mode, mobile phase A comprised 5% acetonitrile with 0.1% formic acid in water and mobile phase B was 95% acetonitrile with 0.1% formic acid in water. For negative ion mode, mobile phase A comprised 5% acetonitrile with 1 mM ammonium fluoride in water and mobile phase B was 95% acetonitrile with 1 mM ammonium fluoride in water. The following gradient was used: 0 min – 0% B; 1 min – 0% B; 5 min – 20% B; 20 min – 100% B; 30 min – 100% B; 30 min 30 sec – 0% B; 34 min – 0% B, 2 min recalibration post-time. The flow rate was 0.25 mL min^{-1} and the column temperature was held at 35 °C for the duration. The source conditions for electrospray ionization were as follows: gas temperature was 325 °C with a drying gas flow rate of 9 L min^{-1} and a nebulizer pressure of 35 psig. The capillary voltage was 3250 V in both positive and negative ion mode. The fragmentor voltage was 115 V and skimmer 70 V. Scanning was performed using the autoMS/MS function starting with a survey scan with a mass range of 50 – 3000 m/z at 4 scans s^{-1}, followed by the MS/MS scan with a mass range of 25 – 3200 m/z at 3 scans s^{-1} with a sloped collision energy of 3.5 V/ 100 Da with an offset of 5 V.

Data extraction and processing

The raw data files (Agilent *.d) of leaf disc-containing samples were processed with Mass Profiler (Version B.08.00, Agilent, Palo Alto, CA, USA) to extract features of interest (FOIs) using the built-in molecular feature extraction algorithm. This process identifies compounds as co-eluting adducts (H^+, Na^+, K^+, NH_4^+) using a 15 ppm mass tolerance and a 0.3 min retention time tolerance. The retention times of features were aligned across samples. This analysis was carried out for samples analysed in positive and negative ion mode and the

resulting combined 5359 features (including predicted neutral mass, molecular formula, retention time and abundance) were exported as text files to MassProfiler Professional (Agilent) for statistical analysis. Log_2 abundance values were percentile (value 75) normalised and baselined to the median of all samples.

Features of interest were exported to MassHunter ID Browser (version B 08.00, Agilent) for generation of predicted molecular formulae based on accurate mass and isotope abundance and searching of an in-house Arabidopsis compound database constructed from AraCyc (https://www.plantcyc.org/databases/aracyc/16.0). Further annotation of FOIs was carried out by accurate mass searches in MassBank (http://www.massbank.jp/), Metlin (https://metlin.scripps.edu/landing_page.php?pgcontent=mainPage), MZCloud (https://www.mzcloud.org/), Human Metabolome Database (http://www.hmdb.ca/), LipidBank (http://lipidbank.jp/) and FooDb (http://foodb.ca/).

References to Methods S1

Barth C, Jander G (2006) Arabidopsis myrosinases TGG1 and TGG2 have redundant function in glucosinolate breakdown and insect defense. Plant J. doi: 10.1111/j.1365-313X.2006.02716.x

Bednarek P, Piślewska-Bednarek M, Svatoš A, Schneider B, Doubský J, Mansurova M, Humphry M, Consonni C, Panstruga R, Sanchez-Vallet A, et al (2009) A glucosinolate metabolism pathway in living plant cells mediates broad-spectrum antifungal defense. Science (80-). doi: 10.1126/science.1163732

Bender J, Fink GR (1998) A Myb homologue, ATR1, activates tryptophan gene expression in Arabidopsis. Proc Natl Acad Sci U S A. doi: 10.1073/pnas.95.10.5655

Ellis C, Turner JG (2001) The Arabidopsis mutant cev1 has constitutively active jasmonate and ethylene signal pathways and enhanced resistance to pathogens. Plant Cell **13**: 1025–1033

Ellis C, Turner JG (2002) A conditionally fertile coi1 allele indicates cross-talk between plant hormone signalling pathways in Arabidopsis thaliana seeds and young seedlings. Planta **215**: 549–556

Noir S, Bömer M, Takahashi N, Ishida T, Tsui T-L, Balbi V, Shanahan H, Sugimoto K, Devoto A (2013) Jasmonate controls leaf growth by repressing cell proliferation and the

onset of endoreduplication while maintaining a potential stand-by mode. Plant Physiol **161**: 1930–51

Smolen G, Bender J (2002) Arabidopsis cytochrome p450 cyp83b1 mutations activate the tryptophan biosynthetic pathway. Genetics

Westphal L, Scheel D, Rosahl S (2008) The coi1-16 Mutant Harbors a Second Site Mutation Rendering PEN2 Nonfunctional. Plant Cell **20**: 824–826
